# Effectiveness of noninvasive brain stimulation techniques on cognitive function in individuals with Alzheimer’s disease and MCI: a systematic review and meta-analysis

**DOI:** 10.3389/fnagi.2026.1782521

**Published:** 2026-07-27

**Authors:** Li-Chin Lu, Shao-Huan Lan, Shou-Jen Lan, Yen-Ping Hsieh

**Affiliations:** 1 Department of Business Administration, Guangzhou Institute of Science and Technology, Guangzhou, China; 2 School of Artificial Intelligence, Guangzhou Institute of Science and Technology, Guangzhou, China; 3 Department of Research, Chanhua Christian Hospital, Changhua, Taiwan; 4 Department of Long-term Care, National Quemoy University, Kinmen, Taiwan

**Keywords:** ADAS-cog, Alzheimer’s disease, global cognition, mini-mental state examination, noninvasive brain stimulation

## Abstract

**Background:**

Noninvasive brain stimulation (NIBS) may alleviate cognitive impairments in individuals with Alzheimer’s disease (AD). However, clinical findings remain inconsistent, highlighting the need for a systematic review and meta-analysis to clarify the efficacy of NIBS.

**Objective:**

To compare the effects of NIBS and sham stimulation on global cognition in individuals with AD.

**Methods:**

Randomized controlled trials were identified from PubMed, Web of Science, Scopus, and the Cochrane Central Register of Controlled Trials by using relevant search terms. Cognitive outcomes were assessed using objective scales, and pooled standardized mean differences with 95% confidence intervals (CIs) were calculated using a random-effects model.

**Results:**

NIBS was associated with significant improvements in global cognition in individuals with AD, as measured using the Mini-Mental State Examination (MMSE; standardized mean difference = 0.58, 95% CI = 0.32 to 0.84, *p* < 0.001) and Alzheimer’s Disease Assessment Scale–Cognitive Subscale (ADAS-Cog; standardized mean difference = −0.40, 95% CI = −0.68 to −0.13, *p* = 0.004). The subgroup analysis revealed that treatment is efficacious when patients are stable medication users for 3 months or less (MMSE scores), intervention is delivered for 6 weeks, and NIBS is administered singularly. Longer duration of pharmacological treatment was associated with reduced NIBS efficacy on MMSE scores in both the meta-regression and subgroup analyses. Cognitive gains based on MMSE outcomes were maintained at 4 and 8 weeks after treatment.

**Conclusion:**

This meta-analysis provides evidence for the effects of NIBS on common cognitive outcomes in AD and explores potential moderators related to sample and intervention characteristics.

**Systematic review registration:**

https://www.crd.york.ac.uk/PROSPERO/view/CRD420251065634.

## 1 Introduction

According to the World Health Organization, more than 50 million individuals were living with dementia in 2019; this number is expected to increase to 139 million in 2050 (World Health Organization, 2021). The financial burden associated with dementia is also likely to more than double from the US$1.3 trillion per year recorded in 2019 to US$2.8 trillion by 2030 (Alzheimer's Disease International, 2023). Neurocognitive disorders, including mild cognitive impairment (MCI) and dementia, are a challenge not only for those affected but also for their families. Because of a set of cognitive impairment-related symptoms involving both functional decline and increased dependency, caring for individuals with dementia and cognitive impairment is associated with a high financial and care burden. Family caregivers are required to care for individuals with dementia and handle unexpected situations such as behavioral problems and changes in patient characteristics; studies have indicated that in general, 57% of unpaid caregivers of individuals with Alzheimer disease (AD) provide care for an average of 9 h per day over ≥4 years (Kasper et al., 2015; Fisher et al., 2011). As dementia progresses, each instance of a decrease in physical activity function can result in nearly 5 additional hours of monthly caregiving. Caregivers often experience increased care burdens, stress, and negative emotions or mental health problems (eg, depression) due to the duration of care (Van der Lee et al., 2014). In consideration of this, effective intervention strategies that mitigate dementia progression and alleviate the major physical and psychological problems related to the caregiving process must be identified.

Scientific and medical advancements have enabled research on novel, innovative technological care models. For example, noninvasive brain stimulation (NIBS) techniques can be used to induce neuronal plasticity in the brain through the modulation of cortical neuron excitability (Huang et al., 2017). Non-invasive brain stimulation (NIBS) techniques, including repetitive transcranial magnetic stimulation (rTMS) and transcranial direct current stimulation (tDCS), rTMS induces neuronal firing through repetitive magnetic pulses (Klomjai et al., 2015), whereas tDCS alters resting membrane potentials using weak direct electrical currents (Chase et al., 2020; Kronberg et al., 2017). Through long-term potentiation (LTP)- and long-term depression (LTD)-like mechanisms, both approaches have demonstrated potential benefits for memory and cognitive function in individuals with cognitive impairment. Compared with tDCS, rTMS directly evokes neuronal activity and may produce more focal neuromodulatory effects, whereas tDCS primarily modulates neuronal excitability in a simpler and more portable manner (Klomjai et al., 2015; Chase et al., 2020; Kronberg et al., 2017). Although a meta-analysis revealed that NIBS efficacy in 6 cognitive domains of patients with Parkinson disease (PD) may be limited (Giustiniani et al., 2025), another meta-analysis reported that rTMS and tDCS significantly improved memory functions in patients with mild cognitive impairment (MCI), with rTMS having a more substantial effect than that of tDCS (Hu et al., 2024). In addition, in their meta-analysis, Teselink et al. reported that NIBS techniques significantly improve global cognition but that most subgroup analyses on their cognitive function effects have focused on rTMS and patients with AD, without considering tDCS or patients with MCI (Teselink et al., 2021). In total, 11 meta-analyses on the effects of rTMS in patients with AD have reported significant improvements in global cognitive function (Alcalá Lozano et al., 2017; Chou et al., 2020; Dong et al., 2018; Li et al., 2024; Liao et al., 2015; Lin et al., 2019; Menardi et al., 2022; Terao and Kodama, 2025; Wang et al., 2020; Xue et al., 2024; Zhang et al., 2025). Only one study revealed no significant improvement in cognition, as measured using the Mini-Mental State Examination (MMSE) (Dong et al., 2018). Three meta-analyses on the effects of tDCS in patients with AD indicated a significant improvement in overall cognition (Chen et al., 2022; Hou et al., 2024; Majdi et al., 2022). One study, however, reported that these effects did not remain significant over the long term (Li et al., 2024). In general, clinical trials have provided inconsistent results with respect to the effects of NIBS techniques on cognitive outcomes in patients with AD.

NIBS techniques may also be used in combination with various nonpharmaceutical modalities, such as cognitive training (CT) and aerobic exercise, to achieve a synergistic effect. CT involves a set of standard tasks designed to reflect specific cognitive functions involving cognitive domains such as memory, attention, and executive processing, with the tasks tailored to an individual’s level of ability (Woods et al., 2023). It can be delivered to an individual or group, with training on pencil and paper or through the Internet (Woods et al., 2023). A meta-analysis focused on NIBS combined with CT revealed a significant positive short-term effect of tDCS combined with CT in patients with MCI and AD (Chu and Cheng, 2025). Another meta-analysis on NIBS combined with CT reported a significant positive immediate effect of rTMS combined with CT in patients with MCI and AD on the Alzheimer’s Disease Assessment Scale-Cognitive Subscale (ADAS-Cog) and MMSE; over the follow-up period, the positive effect was retained on the ADAS-Cog but not on the MMSE (Liu et al., 2023). Thus, additional controlled studies providing robust, evidence-based insights into the effectiveness of NIBS combined with CT interventions are warranted. Moreover, the synergistic effects of NIBS and CT on the cognition of patients with AD in both the short and long term should be assessed.

Individuals with cognitive impairment primarily require maintenance of a daily routine. NIBS techniques are noninvasive and generally well tolerated, with few reported adverse effects (Rossi et al., 2009; Bikson et al., 2016). They can be used alone or in combination with cognitive training (CT) (Chu and Cheng, 2025; Liu et al., 2023). These techniques represent a highly inclusive, supportive option for individuals with Alzheimer’s disease (AD), including those with prodromal or early-stage AD. This study specifically focuses on populations in the AD spectrum. However, the efficacy of NIBS techniques varies with multiple factors, including demographic characteristic (age, disease stage, and medication usage), implementation characteristics (stimulation type and target area, adjunctive CT, and training delivery model [home or laboratory based]), intervention regimen characteristics (length, duration, frequency, and exposure time), and outcome measure parameters (global cognition and cognitive domains). In the current meta-analysis, we evaluated the effects of NIBS techniques, both alone and in combination with CT, in individuals with AD and MCI, as well as the retention time of their impact on global cognition. We also elucidated the optimal treatment protocol, considering different intervention parameters, sample characteristics, and cognitive function. In addition, we summarized commonly used intervention parameters and explored potential factors associated with treatment effects, including intervention characteristics, sample features, and cognitive domains. Our results may aid future randomized controlled trials (RCTs) in generating robust evidence corroborating the efficacy of NIBS techniques.

## 2 Methods

### 2.1 Study search and selection

Four electronic databases, namely, PubMed, Web of Science, Scopus, and the Cochrane Central Register of Controlled Trials, were systematically searched for relevant articles published before June 22, 2025. Our search strategy included the use of keywords related to the target population (eg, dementia and AD), intervention types (eg, rTMS, tDCS, TMS, and tES), and study design (eg, RCT; see Supplementary 1: Table 1 Search strategy). Additional potentially eligible articles were identified through a manual search of relevant reviews.

We aimed to obtain a complete review of all research on implementing NIBS techniques for individuals with dementia on cognitive function. Thus, to ensure all relevant information was captured, articles were assessed for inclusion through full-text screening. This study adhered to the Preferred Reporting Items for Systematic Reviews and Meta-Analyses guidelines (Page et al., 2021), and the study protocol has been registered with PROSPERO (CRD 420251065634).

### 2.2 Eligibility criteria and outcome measures

We only included articles reporting RCTs on NIBS techniques for patients with AD and MCI. Studies meeting the following criteria were included in our analysis: (1) inclusion of participants with a confirmed clinical diagnosis of AD probable AD; early-stage AD; or AD-related cognitive impairment based on Diagnostic and Statistical Manual of Mental Disorders (DSM), NINCDS-ADRDA Alzheimer’s Criteria, or Clinical Dementia Rating (CDR) criteria established by a physician or by using a validated diagnostic instrument, (2) use of an element of NIBS (a) alone or (b) in combination with CT as the primary intervention, (3) inclusion of comparison groups (controls included, usual care, or sham interventions), (4) measurement of study outcomes by using standardized assessment instruments and at least one described outcome targeting cognitive function, and (5) an RCT study design. Studies meeting the following criteria were excluded: (1) inclusion of (a) healthy individuals or patients with other psychiatric or neurological conditions (e.g., major depressive disorder, severe brain injury, other neurological disorders, or coma), (b) patients with dementia types other than AD, such as dementia with Lewy bodies, frontotemporal dementia, vascular dementia, or Parkinson’s disease, or (c) animal models; (2) inclusion of interventions involving stimulation through photobiomodulation or low-level laser therapy, (3) administration of any form of NIBS (eg, rTMS, tDCS, or a similar modality) to the control group, (4) lack of data pertaining to the outcome of interest, (5) a study design other than RCT (case report, case series, protocol, poster or conference abstract, observational study, or review), and (6) publication only in a non-English language.

The titles and abstracts of the identified article records were first imported into EndNote, and 2 investigators (LLC and LSH) independently screened and reviewed each study. When any disagreement occurred, a third investigator (HYP) was consulted to reach a consensus. The following data were extracted from each study: year of publication, study design, number of participants, intervention plan, and study outcomes. Study-targeted global cognitive function was considered the primary outcome endpoint (eg, MMSE, MoCA, and ADAS-Cog scores); follow-up outcomes and several other cognitive domains (ie, memory, language, comprehension, praxis, orientation) were regarded as secondary outcomes.

### 2.3 Data analysis

We extracted the mean change scores from baseline (primary metric) and their standard deviations, as well as the numbers of participants in each treatment group at each assessment point. Jruler was used to extract data available only in figure format. Cochrane Risk of Bias 2.0 was used to evaluate the quality and risk of bias in the included RCTs (Sterne et al., 2019). Statistical analyses were conducted using a random-effects model (DerSimonian and Kacker, 2007) in Review Manager (version 5.3; Nordic Cochrane Centre, Copenhagen, Denmark), and treatment efficacy and publication bias were estimated using Comprehensive Meta-Analysis (version 3.0; Biostat, Englewood, NJ, USA). Standardized mean differences were calculated using Hedges’ *g* to correct for small-sample bias. The degree of heterogeneity was evaluated using *Q* statistics generated from the χ^2^ test and *I*^2^ statistics. Subgroup analyses by NIBS modality were conducted as an exploratory (*post hoc*) method to investigate potential sources of heterogeneity. The results of these analyses were interpreted with extra caution. A random-effects model was used to calculate the mean difference, and 95% CI for outcome analysis. The Begg test was performed to determine the correlation between the ranks of effect sizes. The Egger regression asymmetry test was employed to analyze the association between the estimated intervention effects and the measured sample size. Meta-regression analysis was used to evaluate the effects of 6 intervention time variable types: total intervention period (number of weeks), total number of intervention sessions (number of sessions per week × number of weeks), total intervention time (number of sessions × length of each session × number of weeks), weekly intervention frequency (number of weekly sessions), length of each intervention session, and intervention exposure time per week (number of weekly sessions × length of each session).

## 3 Results

### 3.1 Study selection

**Figure 1:**
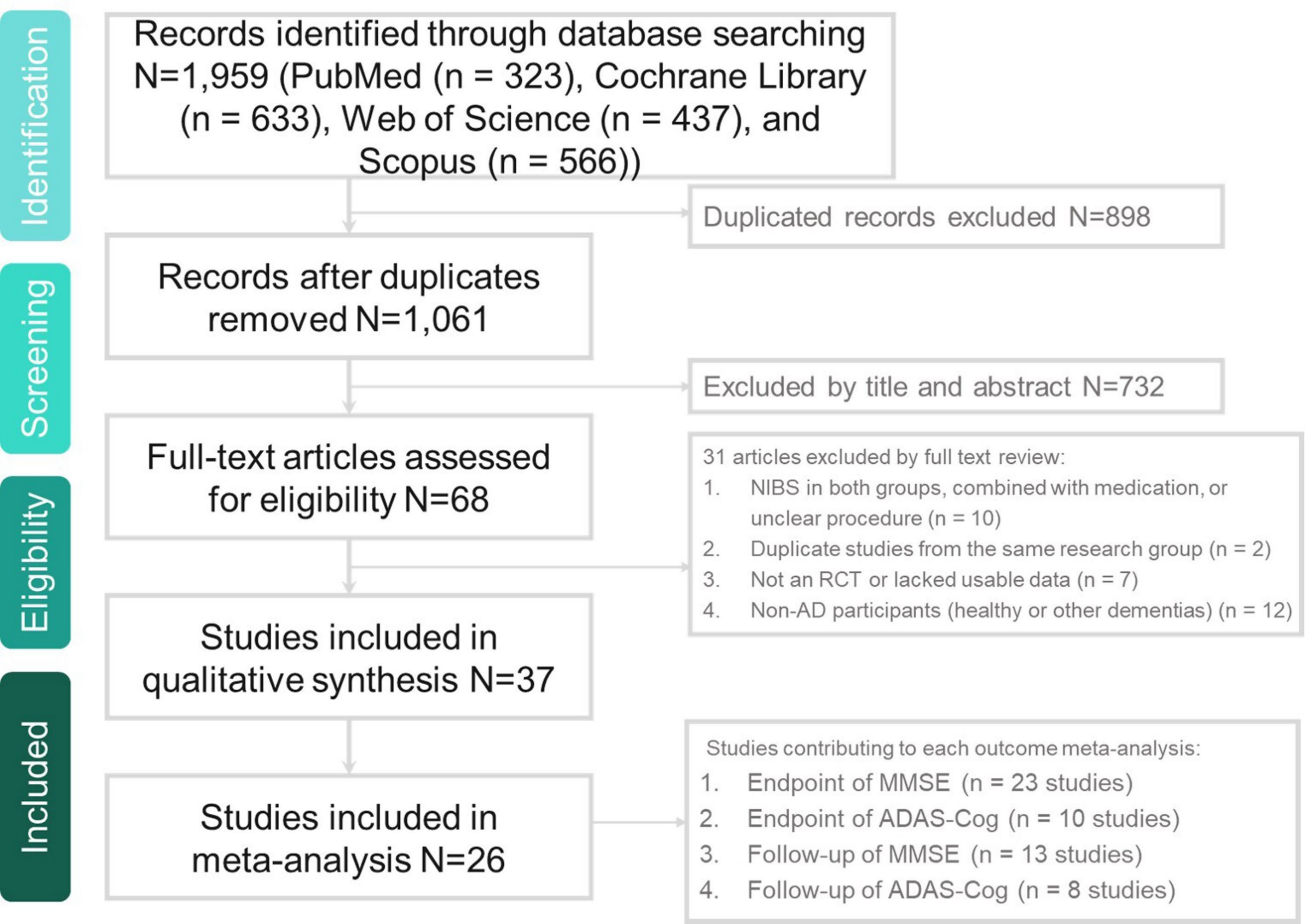
Flowchart of the study selection process.

Our search initially yielded 1,959 articles, including 323 from PubMed, 633 from Cochrane Library, 437 from Web of Science, and 566 from Scopus. From these articles, 898 duplicates were excluded. The remaining 1061 articles were subjected to title and abstract screening. Of the 68 articles that were subsequently retained, 31 were excluded for the following reasons: (1) NIBS was applied in both groups (*n* = 7), the experimental group also received medication (*n* = 1), and the intervention process was unclear (*n* = 2); (2) articles reported duplicate studies from the same research group (*n* = 2); (3) the study used a non-RCT design (*n* = 1) or lacked available outcome data (*n* = 6); (4) the participants were healthy (*n* = 1) or had non-AD dementia, such as dementia with Lewy bodies; prominent features of behavioral variant frontotemporal dementia, and HIV-associated neurocognitive disorder; (*n* = 11). Finally, 37 RCTs were included in our qualitative analysis (Figure 1; Supplementary 1: Table 2).

### 3.2 Study characteristics

Tables 1, 2 lists the characteristics of the included RCTs. Of them, 2 were crossover trials (Uehara et al., 2025; Wang et al., 2024), and the remaining were single-unit RCTs (Ahmed et al., 2012; Andrade et al., 2022; Bagattini et al., 2020; Cotelli et al., 2014; Fonte et al., 2024; Hu et al., 2022; Im et al., 2019; Inagawa et al., 2019b; Jia et al., 2021; Khedr et al., 2014; Khedr et al., 2019; Kim and Yang, 2023; Koch et al., 2025; Koch et al., 2022; Lee et al., 2016; Leocani et al., 2020; Li et al., 2023; Li et al., 2021; Lin et al., 2024; LoBue et al., 2025; Meléndez et al., 2023; Moussavi et al., 2024; Qin et al., 2023; Rabey et al., 1996; Saitoh et al., 2022; Satorres et al., 2023; Suemoto et al., 2014; Tang et al., 2024; Wei et al., 2022; Wu et al., 2022; Wu et al., 2024; Yao et al., 2022; Zhang et al., 2019; Zhao et al., 2017; Zhou et al., 2022). Between 2012 and 2025, a total of 37 RCTs were published in various Western and non-Western countries (region). Studies from Western countries were from the following countries: Italy (5 studies), Canada (2 studies), Spain (2 studies), the United States (2 studies), and Israel (1 study). Studies from non-Western countries were from the following countries: included China (14 studies), Egypt (3 studies), South Korea (3 studies), Brazil (2 studies), Japan (2 studies), and Taiwan (1 study). A total of 17 RCTs included patients with AD from three birth cohorts (1928–1945, 1946–1964, and 1965–1980), which were grouped according to commonly used U. S. generational classifications. In particular, 3 and 5 RCTs participants from the 1928–1945 and 1946–1964, respectively; moreover, 5 included participants from the 1946–1964 and 1965–1980, whereas 4 included those from the 1928–1945 and 1946–1964. Among the 37 total RCTs, 3 included patients with amnesic MCI (aMCI), 1 included patients with aMCI to moderate AD, 4 included patients with mild AD, 3 included patients with moderate AD, 23 included patients with mild-to-moderate AD, 2 included patients with mild-to-severe AD, and 1 included patients without a specific dementia stage. Finally, the dropout rates were 11 to 20% in 12 RCTs, >20% in 3 RCTs, and 0 to 10% in 22 RCTs.

**Table 1:** Characteristics of included studies.

Author, year	Country, area	Study design	Birth cohort	Medication status simplified description	Stable medication history	Participation characteristics	Follow-up	Intervention/Control sample size,	Age mean (SD), y	N Male/Female
Ahmed et al. (2012)	Egypt	A single-blind, sham-controlled, RCT	1938–1952	At recruitment, no patients had used cholinomimetics, antidepressants, neuroleptics, or sedatives in the past 2 weeks.	N/A Not applicable	Mild to severe AD NINCDS-ADRDA, MMSE = 6–21	1-month follow-up 3-month follow-up	rTMS with 20 Hz 15 rTMS with 1 Hz 15 sham stimulaiton 15	65.9 ± 5.9 68.6 ± 6.7 68.3 ± 4.9	*N* = 45 16/29
Andrade et al. (2022)	Brazil	A double blind RCT	1940–1951	Use of AChEIs and/or NMDA antagonists for > = 3 months before screening.	AChEIs and/or NMDA > =3 months	Mild to moderate AD NINCDS-ADRDA CDR = 1–2, MMSE>12	None	Anodal tDCS +CS 18 sham tDCS+CS 18	75.4 ± 4.777.1 ± 5.2	*N* = 36 19/17
Bagattini et al. (2020)	Italy	A double-blind, sham-controlled RCT	1943–1951	Patients on cholinesterase inhibitors (donepezil or rivastigmine required a stable dose for > = 3 months before study entry).	Cholinesterase inhibitors (donepezil or rivastigmine) > =3 months	aMCI or mild to moderate probable AD MMSE> = 16, CDR = 0.5–2	2-month follow-up	rTMS with 20 Hz + CT 27 sham rTMS+CT 23	73.56 ± 4.91 73.35 ± 1.09	*N* = 50 29/21
Cotelli et al. (2014)	Italy	A double-blind, sham-controlled RCT	1931–1946	All patients had maintained a stable dose of donepezil or rivastigmine for > = 6 months before the study.	Donepezil or rivastigmine > =6 months	Mild to moderate AD, NINCDS-ADRDA,	3-month follow-up 6-month follow-up	Anodal tDCS + IC memory training 12 sham tDCS + IC memory training 12 anodal tDCS + motor training 12	76.6 ± 4.6 74.7 ± 6.1 78.2 ± 5.2	*N* = 36 7/29
Fonte et al. (2024)	USA	A double-blind, sham-controlled RCT	1933–1957	Stable AChEI treatment (e.g., donepezil, rivastigmine) with no changes in the past 4 months.	AChEI treatment (e.g., donepezil, rivastigmine) 4 months	Mild or moderate AD MMSE>15,	1-week follow-up	Anodal tDCS + (MotA) 6 anodal tDCS + (CogA) 7 sham tDCS + (MotA) 6 sham tDCS + (CogA) 4 motor activity (MotA) cognitive activity (CogA)	85 ± 5 78 ± 11 88 ± 3 81 ± 5	*N* = 23 9/14
Hu et al. (2022)	China	A sham-controlled RCT	1937–1952	Patients on memantine and ChEIs for > = 90 days before enrollment were required to maintain a stable dose during the study.	Memantine and ChEIs > = 90 days	Moderate AD 2011 NIA-AA CDR = 2, MMSE = 10-20, GDS < =8, HIS<4, > = 5th-grade education	8-week follow-up	rTMS-tDCS 21 single-tDCS 21 single-rTMS 21 sham stimulation 21	79.33 ± 6.24 77.10 ± 6.88 76.86 ± 6.07 75.33 ± 5.73	*N* = 84 38/46
Im et al. (2019)	South Korea	A double-blind, sham-controlled RCT	1939–1957	All patients were received 5 mg/day donepezil during the study.	Donepezil study duration	Early-stage probable AD, DSM-IV, NINCDS-ADRDA, CDR = 0.5–1	None	Anodal tDCS 11 sham tDCS 7	71.9 ± 9.2 74.9 ± 5.0	*N* = 18 3/15
Inagawa et al. (2019a, 2019b)	Japan	A sham-controlled RCT	1936–1950	Subjects on a stable dose of ChEIs or memantine for > = 2 weeks before enrollment.	ChEIs or memantine > =2 weeks	Mild or major neurocognitive disorder, DSM-5 Pt were excluded from MMSE<18, CDR-J > 2	3-week follow-up	Anodal tDCS + cognitive training task 7 sham tDCS + cognitive training task 13	76.6 ± 5.7 76.2 ± 7.7	*N* = 20 10/10
Jia et al. (2021)	China	A double-blind, sham-controlled RCT	1941–1955	Patients on stable medications for >3 months maintained the same drugs and doses during the 2-week rTMS sessions.	Stable medications > 3 months	Mild to moderate AD DSM-V CDR = 0.5–2	None	Single-rTMS 35 sham stimulation 34	71.41 ± 8.85 73.41 ± 7.73	*N* = 69 21/48
Khedr et al. (2014)	Egypt	A double-blind, RCT	1939–1953	All patients had taken memantine 10 mg/day for > = 3 months before the study.	Memantine 10 mg/day > =3 months	Mild to moderate AD NINCDS-ADRDA	1-month follow-up 2-month follow-up	Anodal tDCS 11 cathodal tDCS 12 sham tDCS 11	68.5 ± 7.2 70.7 ± 5.4 67.3 ± 5.9	*N* = 34 19/15
Khedr et al. (2019)	Egypt	A double-blind, sham-Controlled RCT	1950–1958	All patients received stable treatment for > = 2 weeks before tDCS (memantine 10 mg/d, aspirin 100 mg/d, piracetam 800 mg bid).	Memantine 10 mg/d, aspirin 100 mg/d, piracetam 800 mg bid > =2 weeks	Mild to moderate AD, NINCDS-ADRDA uncertain AD level.	None	Anodal tDCS 23 sham tDCS 21	64.22 ± 3.64 65.23 ± 4.52	*N* = 44 26/18
Kim and Yang (2023)	South Korea	A double-blind, sham-controlled RCT	1946–1962	Uncertain	Uncertain	Mild AD by a neurology specialist.	None	Anodal tDCS 8 sham tDCS 8	71.1 ± 6.6 69.9 ± 8.3	*N* = 16 9/7
Koch et al. (2022)	Italy	A sham-controlled RCT	1942–1957	On AChEI treatment for > = 6 months.	AChEI treatment > =6 months.	Probable mild-to-moderate AD, International Working Group recommendations, CDR = 0.5–1, MMSE = 18–26	None	Precuneus -rTMS 25 Sham-rTMS 25	75.0 ± 5.6 72.3 ± 7.2	*N* = 50 24/26
Koch et al. (2025)	Italy	RCT	1946–1959	On AChEI treatment for > = 6 months.	AChEI treatment > =6 months.	Mild-to-moderate AD, International Working Group recommendations, CDR = 0.5–1, MMSE = 18–26	None	PC-rTMS 27 Sham-rTMS 21 the precuneus (PC)	74.0 ± 5.11 71.2 ± 5.11	*N* = 48 21/27
Lee et al. (2016)	South Korea	A double-blind, sham-controlled RCT	1937–1951	Patients maintained unchanged medication from > = 2 months before and throughout the study.	Unchanged medication rom > = 2 months before and throughout the study	Mild-to-moderate AD, DSM-IV, MMSE = 18–26, CDR = 1–2	6-week follow-up	rTMS-COG 18 sham rTMS 8	72.1 ± 7.6 70.3 ± 4.8	*N* = 26 11/15
Leocani et al. (2020)	Italy	A double-blind, sham-controlled RCT	1940–1958	All but two subjects received standard AD treatment at therapeutic doses: rivastigmine (*n* = 7), donepezil (*n* = 9), memantine (*n* = 2), rivastigmine + memantine (*n* = 7), donepezil + memantine (*n* = 2).	Rivastigmine (*n* = 7), donepezil (*n* = 9), memantine (*n* = 2), rivastigmine + memantine (*n* = 7), donepezil + memantine (*n* = 2) uncertain.	Mild-to-moderate AD, NINCDS-ADRDA, MMSE<=24	4-month follow-up	H2-coil rTMS 12 sham rTMS 16	69.6 ± 7.9 72.6 ± 8.3	*N* = 28 15/13
Li et al. (2021)	China	A double-blind, sham-controlled RCT	1948–1964	All patients used the same ChEI, donepezil.	ChEI, donepezil uncertain.	Moderate AD DSM-V, BEHAVE-AD> = 8, MMSE = 10–26	3-month follow-up	Single-rTMS 37 sham stimulation 38	65.97 ± 8.47 64.58 ± 7.88	*N* = 75 44/31
Li et al. (2023)	China	A double-blind, sham-controlled RCT	1942–1954	Uncertain	Uncertain	Mild-to-moderate AD, International Classification of Diseases-11th Edition MMSE<=26	None	Single-tDCS 63 sham stimulation 61	76.71 ± 5.80 75.57 ± 6.11	*N* = 124 41/83
Lin et al. (2024)	China	A double-blind, sham-controlled RCT	1950–1967	Stable donepezil dose for > = 3 months before iTBS and through follow-up.	Donepezil > =3 months	Mild AD NIA-AA CDR = 0.5 or 1, MMSEM<=27	None	The accelerated high-dose iTBS treatment 30 sham stimulation 15	68.67 ± 6.86 64.46 ± 7.73	*N* = 45 16/29
LoBue et al. (2025)	USA	A double-blind, sham-controlled RCT	1942–1965	Most participants were on stable donepezil/rivastigmine (81%) and memantine (57%) for > = 1 month, except one who began memantine the week of 2 mA HD-tDCS.	Donepezil/rivastigmine (81%) and memantine (57%) > =1 month	Probable AD, uncertain AD level.	2-month follow-up	HD-tDCS 1 mA 9 HD-tDCS 2 mA 9 sham tDCS 5	74.0 ± 9.5 68.4 ± 7.8 69.4 ± 9.0	*N* = 23 12/11
Meléndez et al. (2023)	Spain	A single-blind, sham-controlled RCT	1944–1953	Uncertain	Uncertain	Mild-to-moderateAD, NIA-AA, MMSE = 16–22, GDS = 4, Memory Alteration Test <30	1-month follow-up	Anodal tDCS 9 sham tDCS 9	73.7 ± 3.8 75.4 ± 4.4	*N* = 18 7/11
Moussavi et al. (2024)	Canada	A double-blind, sham-controlled RCT	1940–1958	On a stable (or no) AChEI dose for > = 3 months before entry, with no planned changes during the study.	AChEI > =3 months	Mild or moderate AD confirmed by neurologist or psychiatrist, MoCA = 7–25, CDR = 1–2, CSDD<18, A HIS 7 is considered as VaD, while a 4 < HIS < 7is considered as AD-CVD	2-month(12wk) 4-month(20wk) 6-month(28wk)	Real rTMS-2 weeks 52 Real rTMS-4 weeks 53 sham rTMS-4 weeks 51	73.8 ± 8.3 73.3 ± 6.9 75.0 ± 9.1	*N* = 156 85/71
Qin et al. (2023)	China	A double-blind, sham-controlled RCT	1948–1966	Stable donepezil or memantine for > = 3 months.	Donepezil or memantine > =3 months.	Mild or moderate AD NINCDSADRDA CDR = 0.5–2, HIS<4, >6th-grade education	4-week follow-up	Real rTMS-cognitive training (CT) 10 sham rTMS-cognitive training (CT) 6	65.60 ± 8.06 66.50 ± 9.40	*N* = 16 5/10
Rabey et al., 1996	Israel	A double-blind, sham-controlled RCT	1929–1948	Patients on ChEIs and/or memantine for > = 2 months before the study were eligible.	ChEIs and/or memantine > =2 months	Mild to moderate AD, DSM-IV, MMSE = 18–24, CDR = 1	None	rTMS-COG 7 sham treatment 8	72.6 ± 8.9 75.4 ± 9.07	*N* = 15 10/5
Saitoh et al. (2022)	Japan	A double-blind, sham-controlled RCT	1945–1947	Concurrent AD medication or prior use >6 months without cognitive improvement.	Concurrent AD medication or prior use > 6 months	Mild to severe AD, NINCDS-ADRDA A, MMSE<=25, CDR = 1–2	6-month follow-up	rTMS (120% RMT) 15 rTMS (90% RMT) 13 sham rTMS 12	76.2 77.2 75.8	*N* = 40 15/25
Satorres et al. (2023)	Spain	A single -blind, sham-controlled RCT	1942–1956	None	None	mNCD DSM-5 criteria, GDS = 3, MMSE = 18–25,	None	Real tDCS 17 sham tDCS 16	76.6 ± 5.7 73.4 ± 6.2	*N* = 33 17/16
Suemoto et al. (2014)	Brazil	A double-blind, sham-controlled RCT	1924–1941	Aged > = 50 and on stable AChEI doses for > = 2 months before enrollment.	AChEI doses > =2 months	Moderate AD, MMSE = 10–20	None	Anodal tDCS 20 sham tDCS 20	79.4 ± 7.1 81.6 ± 8.0	*N* = 40 12/28
Tang et al. (2024)	China	RCT	1954–1967	Stable ChEI and memantine dose for >6 weeks.	ChEI and memantine > 6 weeks.	Mild AD NIA-AA CDR = 1, >6th-grade education	3-month follow-up	tACS group 23 sham group 23	65.87 ± 5.21 63.70 ± 6.05	*N* = 46 16/30
Uehara et al. (2025)	Canada	A crossover, double-blind, sham-controlled RCT	1940–1960	On a stable or no dementia medication for > = 3 months before the study.	Stable or no dementia medicatio*n* > =3 months	Mild to moderate AD, referrals from family physicians, geriatricians, neurologists MoCA = 5–25	> = 8-week washout period	real Transcranial alternating current stimulation (tACS) + with Cognitive Exercises sham tACS R1S2 group = 21 S1R2group = 21	72.9 ± 8.6 76.3 ± 9.3	*N* = 42 26/16
Wang et al. (2024)	Taiwan	A crossover, double-blind, sham-controlled RCT	1938–1958	Uncertain	Uncertain	Very mild to mild AD, DSM-5, CDR = 0.5–1	None	Anodal tDCS 14 sham tDCS 16	Mean (Min–Max) 76.5 (66–86) 74.9 (67–84)	*N* = 30 12/18
Wei et al. (2022)	China	A double-blind, sham-controlled RCT	1944–1960	Uncertain	Uncertain	Mild-to-moderate AD, DSM-V, CDR = 0.5–2,	3-month follow-up	rTMS group 29 sham group 27	70.00 ± 8.63 71.67 ± 7.16	*N* = 56 16/40
Wu et al. (2024)	China	A assessor -blind, sham-controlled RCT	1947–1963	Twelve weeks post-assessment, a second iTBS session was given; all patients were on stable ChEI doses.	All patients were on stable ChEI doses.	Mild-to-moderate AD, NIA-AA; MMSE = 10–27, CDR = 0.5–2,	None	iTBS group 23 control group 25 (measurement of the rest motor threshold every 13 weeks, but no iTBS treatment)	66.80 ± 8.84 65.32 ± 7.31	*N* = 42 13/29
Wu et al. (2022)	China	RCT	1948–1964	Treated with stable donepezil for > = 3 months before iTBS through follow-up.	donepezil > =3 months	Mild-to-moderate AD, NINCDS-ADRDA, MMSE = 10–27, CDR < =2,	2-month follow-up 8 weeks after cessation of treatment	iTBS group 24 sham group 23	66.46 ± 8.25 66.35 ± 7.99	*N* = 47 21/26
Yao et al. (2022)	China	A double-blind, sham-controlled RCT	1948–1968	Medicated patients had stable ChEI or memantine doses for >90 days.	ChEI or memantine doses >90 days	Mild-to-moderate AD, NIA-AA, >8th-grade education, MMSE>16	2-month follow-up	rTMS group 15 sham group 12	63.87 ± 6.85 67.60 ± 7.88	*N* = 27 14/13
Zhang et al. (2019)	China	A single-blind, sham-controlled RCT	1942–1958	Stable low-dose SSRI use (e.g., sertraline) for > = 3 months before rTMS-CT in 7 sham and 8 real rTMS patients.	Stable low-dose SSRI use (e.g., sertraline) > =3 months	Mild or moderate AD NINCDS-ADRDA	1-month follow-up	rTMS-CT group 15 sham-CT group 13	69.00 ± 8.19 68.54 ± 7.93	*N* = 28 6/22
Zhao et al. (2017)	China	A double-blind, sham-controlled RCT	1941–1953	Patients maintained unchanged medication from > = 2 months before and throughout the study. All received standard AD treatment, including ChEIs and donepezil.	Patients maintained unchanged medication from > = 2 months before and throughout the study.	Mild and moderate AD DSM-IV MMSE = 18–26, CDR = 1–2	6-week follow-up	rTMS group+ cognitive tasks 17 sham group 13	69.3 ± 5.8 71.4 ± 5.2	*N* = 30 13/17
Zhou et al. (2022)	China	A double-blind, sham-controlled RCT	1943–1959	AD medications remained unchanged during the 4-week treatment. No subjects used sleeping drugs within 4 weeks before baseline.	AD medications remained unchanged during the 4-week treatment.	Mild AD NIA-AA, MMSE<=23, MoCA<=23, CDR = 0.5–1, HIS<4, PSQI>8	2-month follow-up	rTMS group 33 sham group 32	Medians (interquartile ranges) 70(64,76) 74(63.25,79)	*N* = 65 21/44

RCT, randomized controlled trial; AD, Alzheimer’s disease; aMCI, amnestic mild cognitive impairment; MCI, mild cognitive impairment; mNCD, mild neurocognitive disorder; VaD, vascular dementia; ChEI, cholinesterase inhibitor; AChEI, acetylcholinesterase inhibitor; NMDA, N-methyl-D-aspartate; MMSE, Mini-Mental State Examination; MoCA, Montreal Cognitive Assessment; CDR, Clinical Dementia Rating; GDS, Global Deterioration Scale; HIS, Hachinski Ischemic Scale; DSM-IV, *Diagnostic and Statistical Manual of Mental Disorders*, Fourth Edition; DSM-5, *Diagnostic and Statistical Manual of Mental Disorders*, Fifth Edition; NIA-AA, National Institute on Aging–Alzheimer’s Association; NINCDS-ADRDA, National Institute of Neurological and Communicative Disorders and Stroke–Alzheimer’s Disease and Related Disorders Association.

**Table 2:** Intervention and efficiency of included studies.

Author, year	Stimulation protocol and targeted area	Regimen of the intervention training delivery at home or lab-based	Global cognition ① Domain-specific cognitive functions ② Memory ③ Attention and Executive function ④ Language ⑤ Visuospatial reasoning ⑥ Praxis Other ⑦Behavioral symptoms	MMSE baseline Mean (SD) Medians (interquartile ranges)	Cognitive function_endpoint Mean (SD) Medians (interquartile ranges)	Disease duration (years) Mean (SD)
Ahmed et al. (2012)	rTMS with 20 Hz rTMS with 1 Hz 20 min 90 mm figure of eight coil the left and right DLPFC	5 days per week, 20-min session or 33-min session, a total of 5 sessions 1 week lab-based	① Global cognitive functioning First group 20 Hz 1-week endpoint outcome: + 1-month follow-up outcome: + 3-month follow-up outcome: + significant differences	rTMS with 20 Hz 14.7 ± 3.7 rTMS with 1 Hz 12.7 ± 3.9 sham stimulaiton 13.9 ± 3.9	MMSE 17.9 ± 5.8 14.2 ± 4.3 13.7 ± 4.1	rTMS with 20 Hz 3.9 ± 2.3 rTMS with 1 Hz 4.1 ± 2.3 sham stimulaiton 4.4 ± 2.5
Andrade et al. (2022)	Anodal tDCS with 2 mA + CS 30 min six brain areas including the right and left dlPFC, Broca’s and Wernicke’s areas, and the right and left pSAC	3 times a week a total of 24 sessions 2 months lab-based	① Global cognitive functioning 2-month endpoint outcome: +	Active tDCS +CS 20.2 ± 0.9 sham tDCS+CS 20.4 ± 1.1	No available data	Months active tDCS +CS 14.78 ± 4.69 sham tDCS+CS 15.01 ± 4.52
Bagattini et al. (2020)	rTMS with 20 Hz + CT 25 min a figure of-eight 70 mm air-cooled coil the left DLPFC	5 days per week, 25-min session with rTMS, 25-min session with CT a total of 20 sessions 4 weeks lab-based	① Global cognitive functioning 4-week endpoint outcome: + 2-month follow-up outcome: + ② Memory ③ Attention ④ Language ⑤ Visuospatial reasoning ⑥ Praxis	rTMS +CT 23.67 ± 3.0 sham rTMS+CT 22.77 ± 0.6	MMSE 24.3 ± 2.4 22.9 ± 3.7	months rTMS +CT 23.33 ± 8.86 sham rTMS+CT 20.09 ± 15.09
Cotelli et al. (2014)	Anodal tDCS with 2 mA + individualized computerized memory training anodal tDCS + motor training 25 min the left DLPFC	tDCS: 5 days per week, 25-min session a total of 10 sessions 2 weeks lab-based	① Global cognitive functioning 2-week endpoint outcome: No significant 3-month follow-up outcome: No significant 6-month follow-up outcome: No significant ② Memory ③ Attention and Executive function ④ Language ⑥ Praxis ⑦ Behavioral symptoms	Anodal tDCS + IC memory training 20.1 ± 2.4 sham tDCS + IC memory training 20.8 ± 2.1 anodal tDCS + motor training 22.1 ± 2.3	MMSE 20.6 ± 2.4 21.7 ± 3.5 22.3 ± 2.4	None
Fonte et al. (2024)	Anodal tDCS with 2 mA + motor activity (MotA) anodal tDCS with 2 mA + cognitive activity (CogA) 25 min the left DLPFC	5 days per week, 45-min session, with the first 15 min using tDCS stimulation a total of 10 sessions 2 weeks lab-based	① Global cognitive functioning 2-week endpoint outcome: + 1-week follow-up outcome: No significant ② Memory ③ Attention ④ Language Other ⑤ Behavioral symptoms	Anodal tDCS + (MotA) 19.6 ± 3.6 anodal tDCS + (CogA) 19.7 ± 4.7 sham tDCS + (MotA) 18 ± 2.5 sham tDCS + (CogA) 20.7 ± 6.2	No available data	Anodal tDCS + (MotA) 4 ± 4 anodal tDCS + (CogA) 3 ± 2 sham tDCS + (MotA) 4 ± 2 sham tDCS + (CogA) 3 ± 4
Hu et al. (2022)	rTMS-tDCS single-tDCS 2 mA single-rTMS 40 Hz 25 min a 70-mm double air diaphragm SHAM Coil The anode was applied to the Angular gyrus (AG) the cathode was applied to the contralateral prefrontal area	3 times a week, one session alternate days, a total of 12 sessions 4 weeks lab-based	① Global cognitive functioning 4-week endpoint outcome: + 8-week follow-up outcome: No significant Other ⑦ Behavioral symptoms	rTMS-tDCS 14.5 ± 3.0 single-tDCS 13.3 ± 3.5 single-rTMS 13.3 ± 2.9 sham stimulation 15.1 ± 3.1	MMSE 19.4 ± 3.6 16.6 ± 3.2 17.1 ± 3.4 15.2 ± 2.9 ADAS-Cog 34.1 ± 9.7 38.6 ± 6.6 36.0 ± 8.9 38.3 ± 10.2	rTMS-tDCS 3.52 ± 1.63 single-tDCS 3.93 ± 1.81 single-rTMS 3.52 ± 1.67 sham stimulation 3.88 ± 2.01
Im et al. (2019)	Anodal tDCS with 2 mA 30 min the left and right DLPFC	One session per day, 7 days per week a total of 168 sessions 24 weeks at home	① Global cognitive functioning 24-week endpoint outcome: + ② Memory ③ Executive function ④ Language ⑤ Visuospatial reasoning	Anodal tDCS 20.1 ± 3.8 sham tDCS 22.1 ± 4.6	MMSE 21.2 ± 4.4 20.6 ± 4.5	None
Inagawa et al. (2019a, 2019b)	Anodal tDCS with 2 mA + cognitive training task 20 min the left DLPFC (F3) and the contralateral supraorbital ridge (Fp2)	5 days per week 20 min per session, two sessions per day, a total of 10 sessions 1 week lab-based	① Global cognitive functioning 1-week endpoint outcome: No significant 2-week follow-up outcome: No significant other ⑧ Attrition rate, FAB, frontal assessment battery	None	None	Anodal tDCS + cognitive training task 0.9 ± 1.2 sham tDCS + cognitive training task 1.2 ± 1.5
Jia et al. (2021)	Single-rTMS with 10 Hz uncertain a figure-of-eight coil (70-mm diameter) the left parietal cortex	5 days per week, one session per day, a total of 10 sessions 2 weeks lab-based	① Global cognitive functioning 2 week endpoint outcome: No significant ② Memory	Single-rTMS 15.7 ± 5.6 sham stimulation 15.6 ± 6.5	MMSE 17.2 ± 6.1 16.2 ± 7.0	None
Khedr et al. (2014)	Anodal tDCS with 2 mA cathodal tDCS with 2 mA 25 min the left DLPFC	Daily for 10 consecutive days, 25 min per session, one session per day, a total of 10 sessions 2 weeks lab-based	① Global cognitive functioning 2-week endpoint outcome: + 1-moth follow-up outcome: + 2-moth follow-up outcome: + Other ⑨ Performance IQ and Verbal IQ scores	Anodal tDCS 18.4 ± 3.9 cathodal tDCS 18.8 ± 2.9 sham tDCS 16.9 ± 2.9	No available data	Anodal tDCS 3.0 ± 2.6 cathodal tDCS 2.9 ± 1.9 sham tDCS 3.5 ± 1.7
Khedr et al. (2019)	Anodal tDCS with 2 mA 20 min the left and right temporo-parietal (TP) lobe	Daily for 10 consecutive days, 20 min per session, one session per day, a total of 10 sessions 2 weeks lab-based	① Global cognitive functioning 2-week endpoint outcome: + ⑤ Visuospatial reasoning	Modified MMSE anodal tDCS 14.2 ± 3.7 sham tDCS 13.9 ± 3.5	Modified MMSE anodal tDCS 17.7 ± 4.6 sham tDCS 13.3 ± 2.8	None
Kim and Yang (2023)	Anodal tDCS with 2 mA 30 min the left and right DLPFC	Daily for 84 consecutive days, 30 min per session, one session per day, a total of 84 sessions 12 weeks at home	① Global cognitive functioning 12-week endpoint outcome: + ② Memory ③ Attention and Executive function ④ Language ⑤ Visuospatial reasoning Other ⑦ The functions related to the frontal lobe	K-MMSE anodal tDCS 23.5 ± 2.8 sham tDCS 22.7 ± 3.5	K-MMSE anodal tDCS 25.6 ± 1.6 sham tDCS 22.2 ± 3.1	Months anodal tDCS 13.5 ± 3.4 sham tDCS 14.4 ± 1.3
Koch et al. (2022)	rTMS with 20 Hz 20 min a 70-mm diameter figure-of-eight coil the precuneus spot	2-week intensive course: consisting of one daily, 20-min session, five consecutive days per week a 22-week maintenance phase in which the same stimulation was applied weekly a total of 32sessions 24 weeks lab-based	① Global cognitive functioning 24-week endpoint outcome: + Other ⑦ Behavioral symptoms ⑧ FAB, frontal assessment battery	PC-rTMS 21.2 ± 2.7 Sham-rTMS 21.5 ± 2.4	MMSE PC-rTMS 20.8 ± 2.8 Sham-rTMS 20.1 ± 1.6	Median (IQR) PC-rTMS 1.4 (0.6–1.9) Sham-rTMS 1.2 (0.4–1.7)
Koch et al. (2025)	rTMS with 20 Hz 20 min a 70-mm diameter figure-of-eight coil the precuneus spot	2-week intensive course: consisting of one daily, 20-min session, five consecutive days per week a 50-week maintenance phase in which the same stimulation was applied weekly a total of 60 sessions 52 weeks lab-based	① Global cognitive functioning 52-week endpoint outcome: + Other ⑦Behavioral symptoms ⑧FAB, frontal assessment battery	PC-rTMS 20.9 ± 2.87 Sham-rTMS 21.9 ± 3.07	No available data	None
Lee et al. (2016)	rTMS with 10 Hz + cognitive training (rTMS-COG) 1 h uncertain six brain areas including the right and left dlPFC, Broca’s and Wernicke’s areas, and the right and left pSAC	5 days per week, Each session lasted 1 h, including preparation, and three brain areas were targeted and stimulated separately. a total of 30 sessions 6 week lab-based	①Global cognitive functioning ADAS-cog 6-week endpoint outcome: + 6-week follow-up outcome: + MMSE 6-week endpoint outcome: + 6-week follow-up outcome: No significant	rTMS-COG 22.4 ± 2.9 sham rTMS 22.8 ± 2.5	MMSE 23.9 ± 4.4 24.5 ± 4.9 ADAS-Cog 19.3 ± 8.3 21.1 ± 7.7	None
Leocani et al. (2020)	H2-coil rTMS with 10 Hz uncertain the frontal–parietal–temporal lobes bilaterally	Treatment consisted in 16 rTMS sessions: 4 weeks of full treatment with 3 sessions/week, followed by 4 weeks with only one weekly maintenance session a total 16 sessions 8 weeks lab-based	①Global cognitive functioning 8-week endpoint outcome: + 4-month follow-up outcome: No significant	H2-coil rTMS 18.0 ± 5.3 sham rTMS 15.5 ± 5.6	ADAS-Cog 28.8 ± 9.2 33.7 ± 9.8	H2-coil rTMS 4.2 ± 2.0 sham rTMS 4.2 ± 1.1
Li et al. (2021)	Single-rTMS with 20 Hz 20 min figure-of-eight coil (Coil- D70-air film coil, Magstim) the left DLPFC	5 days per week, 20-min session, a total of 30 sessions 6 weeks lab-based	①Global cognitive functioning 6-week endpoint outcome: + 3-month follow-up outcome: No significant	Single-rTMS 16.1 ± 0.7 sham stimulation 16.0 ± 0.7	MMSE 18.2 ± 0.6 16.1 ± 0.6 ADAS-Cog 26.2 ± 6.0 28.9 ± 4.8	Single-rTMS 3.70 ± 1.75 sham stimulation 3.97 ± 1.62
Li et al. (2023)	Single-tDCS with 2 mA 20 min the anode placed on the left DLPFC and the cathode on the right DLPFC	5 consecutive days per week stimulated twice a day (once in the morning and once in the afternoon), 20- min session, a total of 30 sessions 6 weeks lab-based	①Global cognitive functioning 6-week endpoint outcome: No significant	Single-tDCS 16.3 ± 4.9 sham stimulation 16.5 ± 4.9	MMSE 18.2 ± 4.4 17.1 ± 4.1 ADAS-Cog 28.6 ± 5.5 33.5 ± 7.7	Single-tDCS 3.83 ± 1.49 sham stimulation 3.90 ± 1.45
Lin et al. (2024)	The accelerated high-dose iTBS uncertain the left DLPFC	Two sessions per day with approximately 50 min intervals a total of 28 iTBS sessions spread over 14 days lab-based	①Global cognitive functioning 2-week endpoint outcome: No significant ②Memory ③Attention and Executive function ⑤Visuospatial reasoning Other ⑦Behavioral symptoms	The accelerated high-dose iTBS treatment 26.6 ± 2.9 sham stimulation 26.8 ± 2.6	MMSE 27.5 ± 3.4 28.3 ± 2.1	The accelerated high-dose iTBS treatment 2.10 ± 2.10 sham stimulation 1.21 ± 0.61
LoBue et al. (2025)	HD-tDCS 1 mA HD-tDCS 2 mA 20 min The anode was placed in the center at Fz (1 or 2 mA intensity) and surrounded by 4 cathodes at FPz, F7, F8, and Cz (each 25% of 1 or 2 mA). the medial prefrontal cortex	5 consecutive days per week, consisting of one daily, 20-min session, a total of 10 sessions 2 weeks lab-based	②Memory ③Attention and Executive function ④Language Other Processing speed	None	None	HD-tDCS 1 mA 3.4 ± 3.3 HD-tDCS 2 mA 1.1 ± 1.0 sham tDCS 2.3 ± 1.0
Meléndez et al. (2023)	Anodal tDCS with 2 mA 20 min the left DLPFC, right frontal lobe	5 consecutive days per week consisting of one daily, 20-min session, a total of 5 sessions 1 week at home	①Global cognitive functioning 1-week endpoint outcome: + 1-month follow-up outcome: + ②Memory ③Attention and Executive function	Anodal tDCS 18.1 ± 1.5 sham tDCS 18.9 ± 2.1	MMSE anodal tDCS 19.0 ± 1.8 sham tDCS 17.6 ± 3.5	None
Moussavi et al. (2024)	rTMS with 20 Hz uncertain air-cooled figure-8 active the left and right DLPFC	Either 2 or 4 weeks of 5 days/week a total of 10 or 20 sessions 4 weeks lab-based	①Global cognitive functioning 4-week endpoint outcome: + 2,4,6-month follow-up outcome: + Other ⑦Behavioral symptoms	MoCA Real rTMS-2 weeks 14.9 ± 4.8 Real rTMS-4 weeks 15.6 ± 5.5 sham rTMS-4 weeks 15.8 ± 4.6	ADAS-Cog 23.4 ± 5.6 23.4 ± 6.9 22.1 ± 5.6	None
Qin et al. (2023)	rTMS with 10 Hz + CT 20 min a butterfly-shaped coil (MCF-B65, inner diameter = 35 mm, outer diameter = 75 mm, winding height = 12 mm) the left DLPFC and then the left LTL	5 times/week. a total of 20 sessions 4 weeks lab-based	①Global cognitive functioning 4-week endpoint outcome: + 4-week follow-up outcome: + ②Memory Other ⑦Behavioral symptoms	Real rTMS-CT 20.2 ± 5.1 sham rTMS-CT 18.3 ± 5.8	MMSE 24.2 ± 5.8 20.6 ± 5.4	Real rTMS-CT 3.1 ± 1.5 sham rTMS-CT 3.7 ± 1.8
Rabey et al. (1996)	rTMS with 10 Hz + COG 45–60 min A 47–86 mm diameter figure of eight magnetic coil six brain areas including the right and left dlPFC, Broca’s and Wernicke’s areas, and the right and left pSAC.	6-week intensive course: consisting of one daily, 45–60 min session, five consecutive days per week 3-month maintenance phase in which the same stimulation was applied bi-weekly a total of 42 sessions (30 + 12) 18 weeks three brain regions were stimulated separately, which stimulated with 400 or 500 pulses over the course of 7–15 min. lab-based	①Global cognitive functioning 18-week endpoint outcome: + Other ⑦Behavioral symptoms	rTMS-COG 22 ± 1.6 sham treatment 22 ± 1.4	No available data	None
Saitoh et al. (2022)	rTMS with 10 Hz uncertain A figure-8-coil the left and right DLPFC	2-week course: four days per week 2-week phase: applied weekly a total of 10 sessions (8 + 2) total 4 weeks lab-based	①Global cognitive functioning 4-week endpoint outcome: + 6-month follow-up outcome: No significant Other ⑦Behavioral symptoms	rTMS (120% RMT) 18.2 ± 4.8 rTMS (90% RMT) 17.7 ± 4.7 sham rTMS 18.6 ± 4.9	No available data	Mean rTMS (120% RMT) 3.5 rTMS (90% RMT) 4.8 sham rTMS 4.6
Satorres et al. (2023)	Active anodal tDCS with 2 mA 20 min The left DLPFC, the right frontal lobe (Fp2)	5 times/week, 20 min. a total of 10 sessions total 2 weeks (10 days) at home	①Global cognitive functioning 2-week endpoint outcome: + ②Memory ③Attention and Executive function ④Language ⑤Visuospatial reasoning ⑥Praxis Other ⑦Behavioral symptoms	Real tDCS 23.88 ± 3.2 sham tDCS 22.94 ± 3.9	MMSE 26.1 ± 3.3 22.5 ± 5.2	None
Suemoto et al. (2014)	Anodal tDCS 20 with 2 mA 20 min the left DLPFC, the right orbit	3 times/week, every other day, 20 min session, a total of 6 sessions 2 weeks lab-based	①Global cognitive functioning 2-week endpoint outcome: No significant Other ⑦Behavioral symptoms	Anodal tDCS 15.0 ± 3.1 sham tDCS 15.4 ± 2.6	No available data	None
Tang et al. (2024)	tACS with 15 mA 1 h A 4.45 × 9.53 cm rectangular pad was positioned on the forehead, aligning with Fpz, Fp1, and Fp2 of the international placement system. four zones, the left and right frontal zone, the left and right posterior zone	Stimulated twice a day (intervals of at least 4 h), 30- min session, five consecutive days per week a total of 30 sessions consecutive 15 days lab-based	①Global cognitive functioning ADAS-Cog: 2-week endpoint outcome: No significant 3-month follow-up outcome: No significant MMSE, MoCA 2-week endpoint outcome: + 3-month follow-up outcome: No significant ②Memory ③Attention and Executive function ④Language Other ⑦Behavioral symptoms	tACS group 19.4 ± 3.3 sham group 20.8 ± 3.0	No available data	None
Uehara et al. (2025)	Real transcranial alternating current stimulation (tACS) with 40 Hz + Cognitive Exercises 30 min uncertain the left DLPFC and contralateral supraorbital area.	5 times/week, (excluding weekends), with two 30 min stimulation paired with cognitive training sessions per day with a 30 min break in between, all in-person. 4 weeks lab-based	①Global cognitive functioning 4-week endpoint outcome: + 8-week follow-up outcome: + Other ⑦Behavioral symptoms	tACS group 22.2 ± 8.9 sham group 21.4 ± 9.7	ADAS-Cog 20.32 ± 8.5 20.23 ± 8.0	None
Wang et al. (2024)	Anodal tDCS with 2 mA 30 min the left DLPFC, the right supraorbital region (RSOR)	5 times/week, 30-min session, a total of 10 sessions 2 weeks after a washout period of 3 months lab-based	① Global cognitive functioning 2-week endpoint outcome: + ③Attention and Executive function	Anodal tDCS 20.3 ± 7.1 sham tDCS 20.9 ± 6.5	MMSE 21.2 ± 6.6 20.3 ± 6.1	Mean (Min–Max) anodal tDCS 3.4 (1–8) sham tDCS 2.9 (1–6)
Wei et al. (2022)	rTMS with 10 Hz 20 min an 8-channel head coil the lateral parietal lobule	Consisting of one daily, 20-min session, 5 consecutive days per week a total of 10 sessions 2 weeks lab-based	① Global cognitive functioning 2-week endpoint outcome: + 3-month follow-up outcome: No significant ②Memory	rTMS group 14.5 ± 6.9 sham group 13.7 ± 7.2	MMSE 15.8 ± 7.3 14.3 ± 7.8	None
Wu et al. (2024)	iTBS with 50 Hz uncertain the left DLPFC	Three cycles of left DLPFC-iTBS on each treatment day, separated by 15 min intervals (total: 1800 pulses/day), 14 consecutive days Treatment was administered every 3 months at intervals determined per a previous study. Within 1 week of the completion of each treatment cycle (T1, T2, T3 and T4) a total of 56 sessions 1-year lab-based	①Global cognitive functioning 1-week endpoint outcome: + ②Memory ③Attention and Executive function ④Language ⑤Visuospatial reasoning Other ⑦Behavioral symptoms	iTBS group 21.4 ± 5.4 control group 21.2 ± 3.9	MMSE 22.7 ± 6.0 17.3 ± 5.9	None
Wu et al. (2022)	iTBS with 50 Hz uncertain the left DLPFC	Three rounds of iTBS on each treatment day (total: 1800 pulses/day, 14 days), three iTBS sessions were separated by 15-min intervals a total of 14 sessions 2 weeks three rounds of iTBS on each treatment day (total: 1800 pulses/day, 14 days). The three iTBS sessions were separated by 15-min intervals 14 consecutive days lab-based	①Global cognitive functioning 2-week endpoint outcome: + 2-month follow-up outcome: + ②Memory ③Attention and Executive function ④Language ⑤Visuospatial reasoning ⑥Praxis Other ⑦Behavioral symptoms	iTBS group 20.5 ± 4.7 sham group 21.7 ± 4.7	MMSE 22.8 ± 5.3 21.0 ± 5.2	None
Yao et al. (2022)	rTMS with 5 Hz 20 min an 8-channel radiofrequency coil bilateral cerebellum	5 times/week, ever day/session, 20 min a total of 20 sessions 4 weeks lab-based	①Global cognitive functioning 4-week endpoint outcome: + 2-month follow-up outcome: + ②Memory ③Attention and Executive function ④Language ⑤Visuospatial reasoning	rTMS group 19.9 ± 4.3 sham group 18.4 ± 5.1	MMSE 23.2 ± 3.6 18.8 ± 4.8 ADAS-Cog 16.9 ± 4.2 19.6 ± 3.1	rTMS group 23.6 ± 15.5 (months) sham group 24.2 ± 15.2 (months)
Zhang et al. (2019)	rTMS with 10 Hz + CT 20 min a focal coil (MCF-B65 Butterfly coil; inner diameter, 35 mm; outer diameter, 75 mm; winding height, 12 mm) the left DLPFC the left LTL	5 times/week, ever day/session, 20 min a total of 20 sessions 4 weeks All patients underwent cognitive training (CT) up to 1 h. The treatment protocol was applied for 4 weeks (5 times/week) and with no maintenance sessions. lab-based	①Global cognitive functioning 4-week endpoint outcome: + 1-month follow-up outcome: + Other ⑦Behavioral symptoms	rTMS-CT group 20.5 ± 4.2 sham-CT group 19.8 ± 5.1	MMSE 23.8 ± 2.2 20.6 ± 1.8	None
Zhao et al. (2017)	rTMS with 20 Hz + cognitive tasks 10 min Uncertain parietal P3/P4 and posterior temporal T5/T6	1 session/day and 5 days/week a total of 30 sessions 6 weeks 10 min of rTMS (10 s of 20 Hz/train, 20s intermediate/train) followed by two to four cognitive tasks were administered over the course of 20–40 s for each brain area. lab-based	① Global cognitive functioning 6-week endpoint outcome: + 6-week follow-up outcome: + ② Memory	rTMS group 22.2 ± 2.8 sham group 22.8 ± 2.3	MMSE 23.9 ± 2.5 23.1 ± 3.3 ADAS-Cog 18.5 ± 5.4 22.9 ± 8.9	None
Zhou et al. (2022)	rTMS with 10 Hz uncertain uncertain the left and right dorsolateral prefrontal lobes	5 times/week, ever day/session, a total of 20 sessions 4 weeks The IG received treatment once a day, five times a week for 4 weeks lab-based	① Global cognitive functioning 4-week endpoint outcome: + 2-month follow-up outcome: + Other ⑦Behavioral symptoms	Medians (interquartile ranges) rTMS group 19(17.636,20.364) sham group 19.094 (17.952,20.235)	No available data	None

ADAS-Cog, Alzheimer’s Disease Assessment Scale–Cognitive Subscale; MMSE, Mini-Mental State Examination; MoCA, Montreal Cognitive Assessment; CT, cognitive training; CS, cognitive stimulation; CogA, cognitive activity; MotA, motor activity; DLPFC, dorsolateral prefrontal cortex; LTL, lateral temporal lobe; TP, temporoparietal cortex; AG, angular gyrus; pSAC, parietal somatosensory association cortex; rTMS, repetitive transcranial magnetic stimulation; tDCS, transcranial direct current stimulation; HD-tDCS, high-definition transcranial direct current stimulation; iTBS, intermittent theta burst stimulation; tACS, transcranial alternating current stimulation; RMT, resting motor threshold; FAB, Frontal Assessment Battery; +:positive effect.

**Figure 2:**
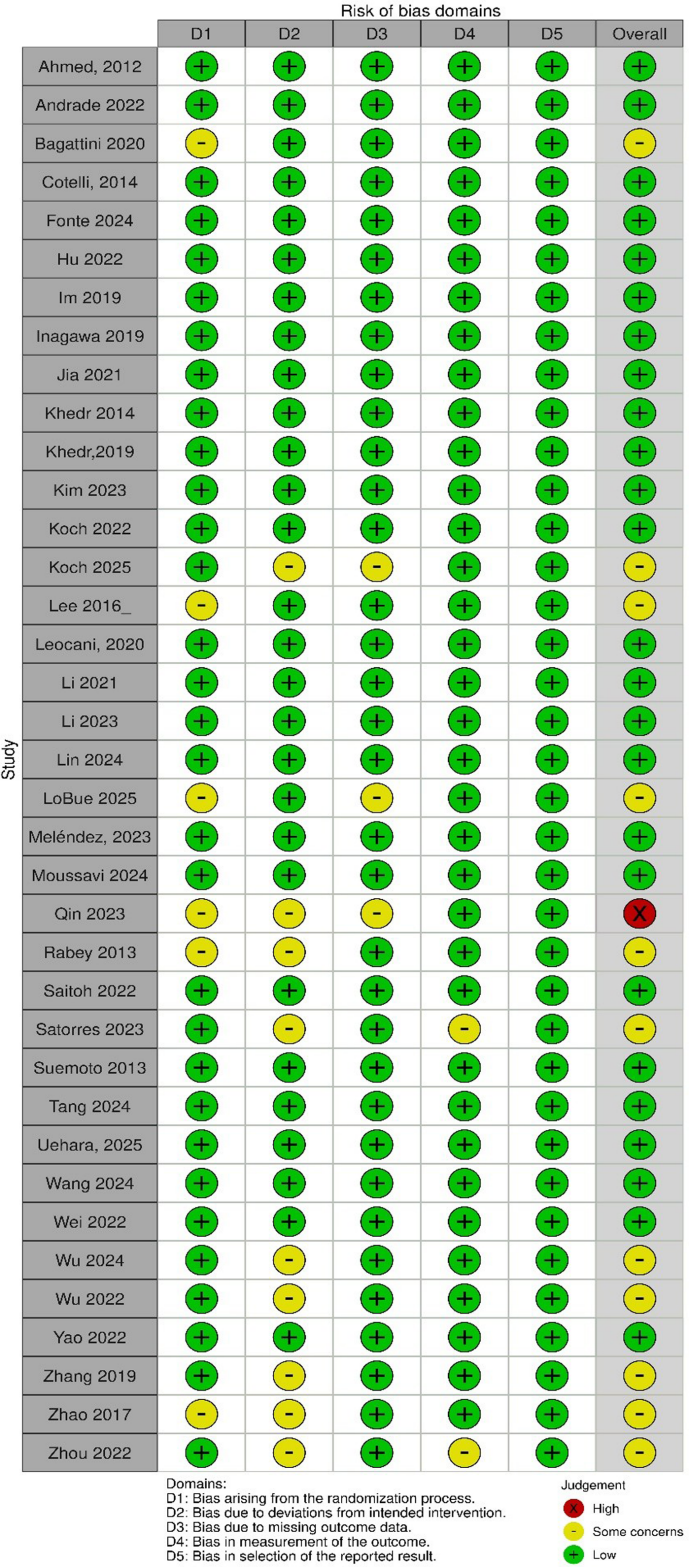
Risk of bias summary.

Figure 2 depicts the evaluation results regarding the risk of bias in each domain for each study. In total, 12 studies (Bagattini et al., 2020; Koch et al., 2025; Lee et al., 2016; LoBue et al., 2025; Qin et al., 2023; Rabey et al., 1996; Satorres et al., 2023; Wu et al., 2022; Wu et al., 2024; Zhang et al., 2019; Zhao et al., 2017; Zhou et al., 2022) demonstrated an overall risk of bias. For 6 studies (Bagattini et al., 2020; Lee et al., 2016; LoBue et al., 2025; Qin et al., 2023; Rabey et al., 1996; Zhao et al., 2017), a risk of bias was present for the randomization process (unclear allocation sequence concealment or randomization). Moreover, in 9 studies, a risk of bias was present for deviation from the intended intervention (lack of clarity regarding the participants (Koch et al., 2025; Qin et al., 2023; Rabey et al., 1996; Zhang et al., 2019; Zhao et al., 2017; Zhou et al., 2022) and trial personnel (Satorres et al., 2023; Wu et al., 2022; Wu et al., 2024; Zhou et al., 2022) being unaware of their assigned intervention during the trial). In 3 studies (Koch et al., 2025; LoBue et al., 2025; Qin et al., 2023), a risk of bias was present for missing outcome data (dropout rate > 20%). Two studies demonstrated a risk of bias related to outcome measurement (lack of clarity regarding the assessors (Satorres et al., 2023; Zhou et al., 2022) being aware of the intervention received by the study participants). In addition, 7 studies (Koch et al., 2025; LoBue et al., 2025; Qin et al., 2023; Rabey et al., 1996; Satorres et al., 2023; Zhao et al., 2017; Zhou et al., 2022) demonstrated a risk of bias pertaining to both deviation from the intended intervention and outcome measurements. Only 1 study (Qin et al., 2023) demonstrated a high overall risk of bias; 25 studies demonstrated a low overall risk of bias in all domains.

Publication bias was examined using funnel plots and evaluated using the Begg and Egger tests. As presented in Supplementary 1: Figures 1a,b, the funnel plots revealed symmetry in the assessments of cognitive function on the MMSE (*p* = 0.43, Egger test; *p* = 0.22, Begg test) and for ADAS-Cog outcomes (*p* = 0.77, Egger test; *p* = 0.55, Begg test).

### 3.3 Primary outcome endpoint

**Figure 3:**
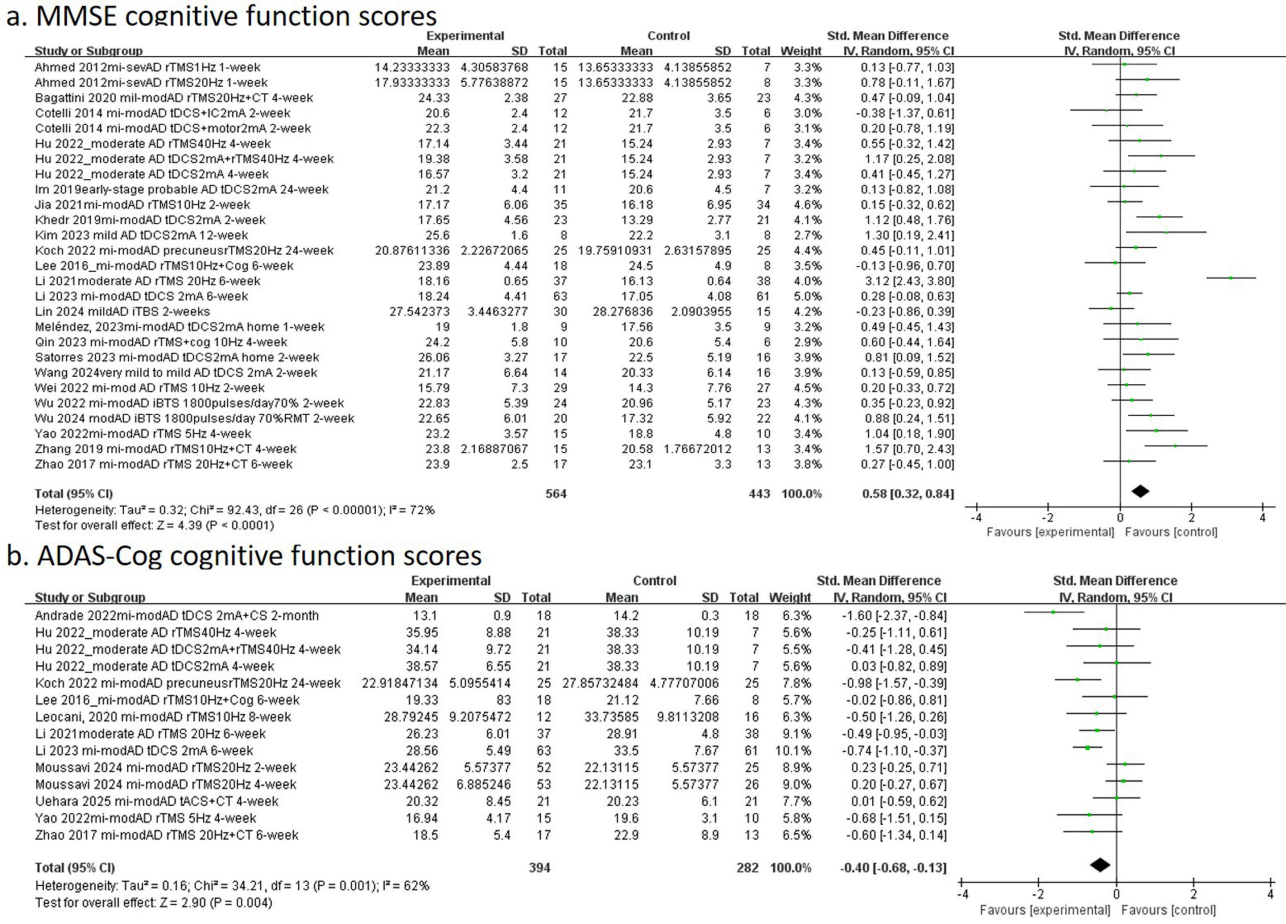
Forest plot of NIBS therapy intervention on cognition.

In total, 23 RCTs (Wang et al., 2024; Ahmed et al., 2012; Bagattini et al., 2020; Cotelli et al., 2014; Hu et al., 2022; Im et al., 2019; Jia et al., 2021; Khedr et al., 2019; Kim and Yang, 2023; Koch et al., 2022; Lee et al., 2016; Li et al., 2023; Li et al., 2021; Lin et al., 2024; Meléndez et al., 2023; Qin et al., 2023; Satorres et al., 2023; Wei et al., 2022; Wu et al., 2022; Wu et al., 2024; Yao et al., 2022; Zhang et al., 2019; Zhao et al., 2017) performed MMSE-based cognitive function analysis and indicated that cognitive function significantly differed between 564 individuals in the NIBS group and 443 in the control group (SMD = 0.58, 95% CI = 0.32 to 0.84, *I*^2^ = 72%, *p* < 0.001; Figure 3a). Subgroup analysis evaluating various forms of NIBS techniques (rTMS, tDCS, rTMS plus tDCS, or intermittent theta burst stimulation (iTBS), which produces facilitatory effects on cortical excitability, either alone or in combination with CT), intervention period (number of weeks), and population characteristics (Western or non-Western ethnicity and stable medication period) was also performed. Among the NIBS techniques, the effect size was relatively larger when rTMS (12 trials; SMD = 0.70, 95% CI = 0.23 to 1.17, *I*^2^ = 83%, *p* < 0.001) or tDCS (9 trials; SMD = 0.45, 95% CI = 0.17 to 0.73, *I^2^* = 28%, *p* = 0.002) was applied alone (Supplementary 2: Figure 1a NIBS technique singularly). Using NIBS as a standalone intervention had a significantly larger effect size than did combining it with CT or other treatments in individuals with AD and MCI (19 trials; SMD = 0.59, 95% CI = 0.28 to 0.89, *I^2^* = 75%, *p* < 0.001; Supplementary 2: Figure 1b NIBS technique combined with CT or not). Regarding intervention periods, the effects sizes were larger when the treatment was delivered over 2 weeks (9 trials; SMD = 0.35, 95% CI = 0.06 to 0.63, *I^2^* = 46%, *p* = 0.02), and 4 weeks (5 trials; SMD = 0.78, 95% CI = 0.46 to 1.10, *I*^2^ = 7%, *p* < 0.001; Supplementary 2: Figure 1c NIBS intervention period) compared with when it was delivered for 1,6 or >6 weeks. Subgroup analysis revealed that studies conducted in non-Western countries had a larger effect size compared with those conducted in Western countries (non-Western countries: 18 trials, SMD = 0.64, 95% CI = 0.32 to 0.96, *I*^2^ = 77%, *p* < 0.001; Western countries: 5 trials, SMD = 0.43, 95% CI = 0.13 to 0.72, *I*^2^ = 0%, *p* = 0.005; Supplementary 2: Figure 1d). These findings should be interpreted with caution because of the heterogeneity within regional categories. Among the analysis of stable medication use duration, our meta-regression result indicated that a longer duration of pharmacological treatment is associated with diminished treatment effects of NIBS (intercept = 3.0621, slope = −0.1046 per week, *p* = 0.0225). The subgroup analysis given similar outcome that greater improvements were observed in participants who received no pharmacological treatment or had been on stable pharmacological treatment for ≤3 months. (3 trials, SMD = 0.61, 95% CI = 0.14 to 1.08, *I*^2^ = 0%, *p* = 0.01; ≤3 months, 13 trials, SMD = 0.52, 95% CI = 0.24 to 0.80, *I*^2^ = 49%, *p* < 0.0003; Supplementary 2: Figure 1e stable medication period; Table 1 subgroup analysis for primary outcome; Supplementary 3: Figure 2 meta-regression of stable medication period). Unspecified stable medication period was observed large effect sizes also (8 trials; SMD = 0.81, 95% CI = 0.11 to 1.50, *I*^2^ = 89%, *p* = 0.02).

In 11 RCTs (Uehara et al., 2025; Andrade et al., 2022; Hu et al., 2022; Koch et al., 2022; Lee et al., 2016; Leocani et al., 2020; Li et al., 2023; Li et al., 2021; Moussavi et al., 2024; Yao et al., 2022; Zhao et al., 2017) including ADAS-Cog cognitive function analysis, compared with the 282 individuals in the control group, the 394 individuals in the NIBS group demonstrated a significant difference in terms of cognitive function (SMD = −0.40, 95% CI = −0.67 to −0.13, *I*^2^ = 62%, *p* = 0.004; Figure 3b). In the subgroup analysis, rTMS alone was associated with a greater effect size than the other techniques were (8 trials; SMD = −0.32, 95% CI = −0.62 to −0.01, *I*^2^ = 53%, *p* = 0.04; Supplementary 2: Figure 2a individual NIBS techniques). Compared with NIBS combined with cognitive training (CT), NIBS administered as a standalone intervention demonstrated a significantly larger effect size in individuals with AD (7 trials; SMD = −0.35, 95% CI = −0.67 to −0.03, *I*^2^ = 63%, *p* = 0.03) (Supplementary 2 Figure 2b NIBS in combination with CT or not). Moreover, compared with <=4-week interventions, the effect sizes were significantly with equal and larger for the 6-week interventions (4 trials; SMD = −0.58, 95% CI = −0.83 to −0.33, *I*^2^ = 0%, *p* < 0.001; 3trials; SMD = −1.03, 95% CI = −1.60 to −1.45, *I*^2^ = 51%, *p* < 0.001; Supplementary 2: Figure 2c NIBS intervention period). The effect sizes for non-Western participants were significantly larger than those for Western participants (7 trials; SMD = −0.57, 95% CI = −0.85 to −0.29, *I*^2^ = 34%, *p* < 0.001; Supplementary 2: Figure 2d population characteristics). Among the three stable medication subgroups, studies with an unspecified stable medication period showed a significant treatment effect (3trials; SMD = −0.62, 95% CI = −0.89 to −0.36, *I*^2^ = 0%, *p* < 0.001; Supplementary 2: Figure 2e stable medication period; Table 1 subgroup analysis for primary outcome).

### 3.4 Secondary outcome follow-up

**Figure 4:**
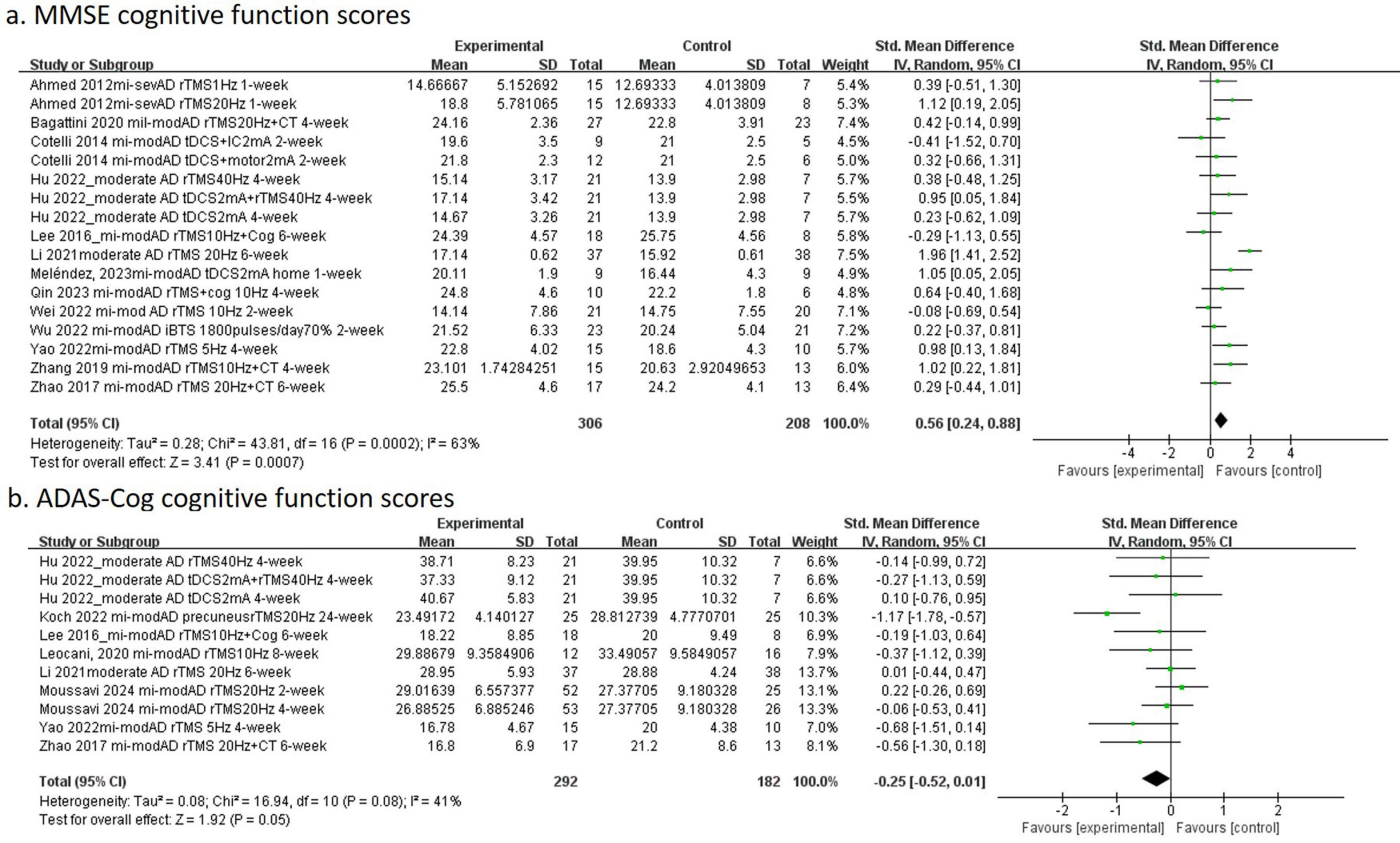
Forest plot of NIBS therapy intervention on cognition in follow up.

In 13 RCTs (Ahmed et al., 2012; Bagattini et al., 2020; Cotelli et al., 2014; Hu et al., 2022; Lee et al., 2016; Li et al., 2021; Meléndez et al., 2023; Qin et al., 2023; Wei et al., 2022; Wu et al., 2022; Yao et al., 2022; Zhang et al., 2019; Zhao et al., 2017) analyzing cognitive function through the MMSE during follow-up, the difference in MMSE cognitive function scores was significant in 306 individuals in the NIBS group compared with in 208 individuals in the control group (SMD = 0.56, 95% CI = 0.24 to 0.88, *I*^2^ = 63%, *p* < 0.001; Figure 4a). In the subgroup analysis, rTMS alone was associated with a greater effect size than the other techniques were (10 trials; SMD = 0.63, 95% CI = 0.19 to 1.07, *I*^2^ = 72%, *p* = 0.005; Supplementary 2: Figure 3a individual NIBS techniques). Compared with NIBS combined with cognitive training (CT), NIBS administered as a standalone intervention demonstrated a significantly larger effect size in individuals with AD (8 trials; SMD = 0.69, 95% CI = 0.22 to 1.16, *I*^2^ = 72%, *p* = 0.004; Supplementary 2: Figure 3b NIBS technique combined with CT or not). In addition, the effects sizes were larger when the treatment was delivered over 4 weeks (3 trials; SMD = 0.93, 95% CI = 0.39 to 1.46, *I*^2^ = 0%, *p* < 0.001) and 8 weeks (4 trials; SMD = 0.47, 95% CI = 0.17 to 0.77, *I*^2^ = 0%, *p* = 0.002; Supplementary 2: Figure 3c NIBS follow-up period). Non-Western ethnicities—in 10 RCTs—were associated with a significantly larger effect size than were Western ethnicities—in 3 RCTs (10 trials; SMD = 0.61, 95% CI = 0.22 to 1.00, *I*^2^ = 69%, *p* = 0.002; 3 trials; SMD = 0.39, 95% CI = −0.09 to 0.86, *I*^2^ = 19%, *p* = 0.11; Supplementary 2: Figure 3d population characteristic). Finally, compared with longer medication periods (6 months, 2 trials; SMD = 0.00, 95% CI = −0.74 to 0.74, *I*^2^ = 0%, *p* = 1.00), a larger effect size was associated with less medication therapy period (none use: 2 trials, SMD = 0.75, 95% CI = 0.10 to 1.40, *I*^2^ = 17%, *p* = 0.02; ≤3 months, 10 trials, SMD = 0.45, 95% CI = 0.20 to 0.69, *I*^2^ = 0%, *p* < 0.001; Supplementary 2: Figure 3e stable medication period).

In 8 RCTs (Hu et al., 2022; Koch et al., 2022; Lee et al., 2016; Leocani et al., 2020; Li et al., 2021; Moussavi et al., 2024; Yao et al., 2022; Zhao et al., 2017) that conducted ADAS-Cog cognitive function analysis during follow-up, the 292 individuals in the NIBS technique group did not differ significantly from the 182 individuals in the control group in terms of cognitive function (SMD = −0.25, 95% CI = −0.52 to 0.01, *I*^2^ = 41%, *p* = 0.05; Figure 4b). Subgroup analysis revealed that rTMS was associated with a larger effect size compared with the other NIBS techniques; however, this difference was not significant (8 trials; SMD = −0.29, 95% CI = −0.59 to 0.01, *I*^2^ = 51%, *p* = 0.06; Supplementary 2: Figure 4a individual NIBS techniques). NIBS alone and combined with CT demonstrated no significant difference in terms of effect sizes during follow-up (Supplementary 2: Figure 4b NIBS technique combined with CT or not). Neither follow-up period nor population characteristics (ethnicity and stable medication period) had significant maintenance effects (Supplementary 2: Figure 4c NIBS follow-up period; Figure 4d population characteristics; Figure 4e stable medication period; Table 2 subgroup analysis for secondary outcome).

### 3.5 Leave-one-out sensitivity and meta-regression analysis

**Figure 5:**
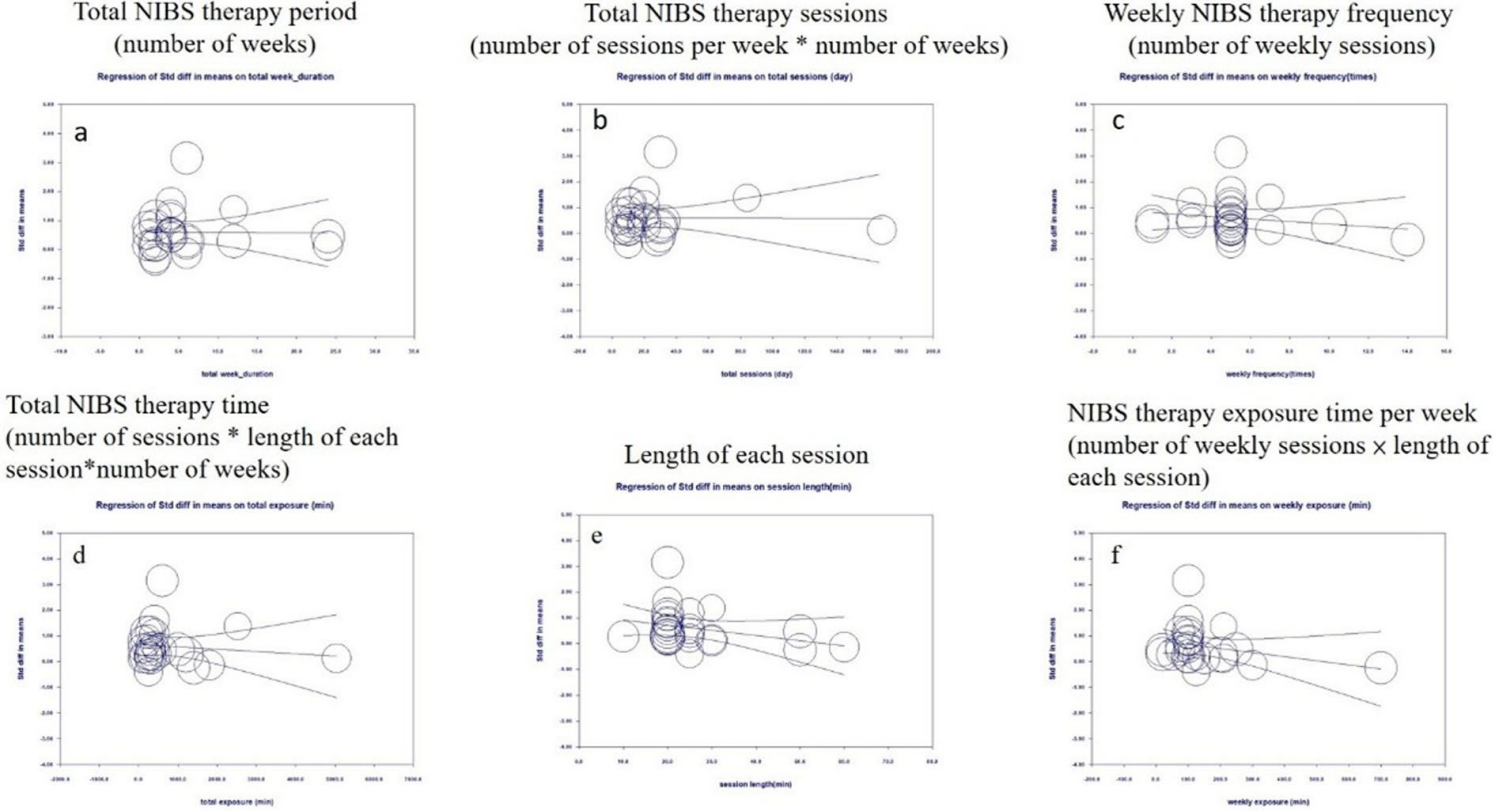
Meta-regression in MMSE scores of 6 types of intervention time.

**Figure 6:**
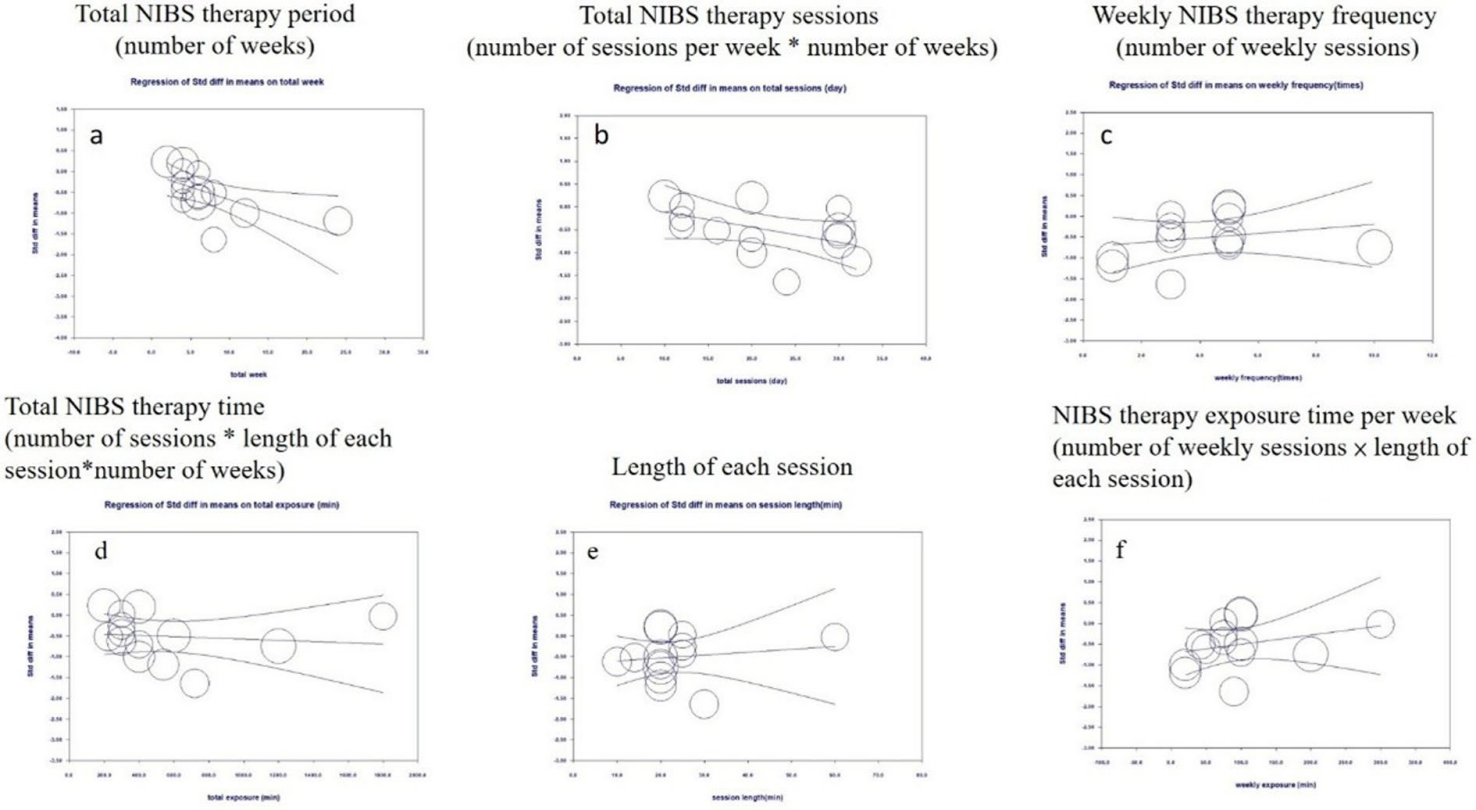
Meta-regression in ADAS-Cog scores of 6 types of intervention time.

Similar preliminary results were obtained in the leave-one-out sensitivity analysis. The new combined effect was compared with the overall combined effect. However, no changes in the results were observed (Supplementary 3: Figures 1a,b leave-one-out meta-analysis). Meta-regression analysis revealed that the 6 time variables had no significant effect on the MMSE cognitive function scores. However, as indicated by the ADAS-Cog scores of these time variables, the total intervention time (*β* = −0.061, *Q* = 7.88, df = 1, *p* = 0.005) and total intervention sessions (*β* = −0.033, *Q* = 3.92, df = 1, *p* = 0.048) mitigated the degree of cognitive impairment (Figures 5, 6; Supplementary 3: Table 1 Meta-regression analyses for MMSE endpoint, ADAS-Cog endpoint).

## 4 Discussion

In this systematic review and meta-analysis of 37 studies, we examined the effects of NIBS on the cognitive function of individuals with AD, including those with prodromal or early-stage AD. Our analysis has 3 major findings that can provide valuable insights into the current state of NIBS interventions. First, various NIBS techniques were associated with improvements of approximately 1.84 points on the MMSE and approximately 2.01 points on the ADAS-Cog, suggesting a modest but potentially clinically meaningful benefit. However, moderate heterogeneity was observed across studies. Meta-regression analysis revealed that intervention characteristics, particularly total treatment duration and total number of sessions, explained part of the between-study variability, with heterogeneity decreasing from 66.92 to 47.30 and 55.73% (*Q* = 39.30 to 22.77 and 27.10), respectively. Nevertheless, residual heterogeneity remained, which suggested the existence of additional sources of variability.

Subgroup analysis further revealed that heterogeneity differed across countries. No heterogeneity was observed for MMSE outcomes in Western countries or for ADAS-Cog outcomes in non-Western countries. The remaining variability may be attributable to differences in educational level, language adaptations of cognitive assessment tools, and baseline population characteristics. Therefore, the pooled results should be interpreted with caution.

Second, several intervention durations (2 and 4 weeks for the MMSE and 6 weeks or longer for the ADAS-Cog) were associated with sustained cognitive benefits. According to the MMSE outcomes, the treatment effects observed at the end of the intervention were maintained at 4 to 8 weeks after treatment.

Finally, population-related study characteristics were associated with a variability in treatment effects. Studies conducted in non-Western countries demonstrated larger effect sizes compared with those conducted in Western countries. However, these regional differences should be interpreted with caution because of the substantial demographic and health-care heterogeneity observed between countries. In addition, a stable medication period of at least 3 months (based on MMSE outcomes) was associated with a major improvement in global cognition after NIBS.

### 4.1 Intervention implementation characteristics [stimulation types, stimulation targeted area, adjunctive CT, and training delivery model (home or laboratory based)]

Our findings indicate that NIBS significantly improved overall cognitive function in patients with early-stage probable AD and mild-to-severe AD, as evaluated using the MMSE (23 trials; SMD = 0.58, 95% CI = 0.32 to 0.84, *I*^2^ = 72%, *p* < 0.001); it also significantly improved overall cognitive function in patients with mild-to-moderate AD, as measured using the ADAS-Cog (10 trials; SMD = −0.44, 95% CI = −0.73 to −0.15, *I*^2^ = 63%, *p* = 0.003). Two previous meta-analyses have reported that active NIBS had positive effects on global cognition in patients with AD and MCI (Hu et al., 2024; Teselink et al., 2021). One meta-analysis, which included 552 patients (N_active_ = 288, N_sham_ = 264) with AD and MCI from 19 studies, reported significant effects of NIBS on global cognition (SMD = 1.14, 95% CI = 0.49 to 1.78, *I*^2^ = 90.2%, *p* = 0.001) (Chase et al., 2020; Kronberg et al., 2017; Giustiniani et al., 2025; Hu et al., 2024; Teselink et al., 2021). In particular, Hu et al. (2024) noted immediate post-rTMS or -tDCS improvement in memory functions in patients with amnestic or nonamnestic MCI (*n* = 202 from 11 studies; SMD = 0.61, 95% CI = 0.41 to 0.82; *p* < 0.00001, *I*^2^ = 22%). Notably, the cognitive function results in both these meta-analyses were obtained using a mixture of scales. Another meta-analysis reported that rTMS or tDCS significantly improved cognition in patients with MCI and AD [measured by combining ≥2 scales in most included studies and by using 1 scale in 1 included study (Liu et al., 2023)]; the studies included in that meta-analysis assessed an NIBS technique either alone [rTMS in 7 meta-analyses (Li et al., 2024; Liao et al., 2015; Lin et al., 2019; Menardi et al., 2022; Wang et al., 2020; Zhang et al., 2025; Fuseya et al., 2025) and tDCS in 3 meta-analyses (Majdi et al., 2022; Li et al., 2024; Muñoz-Perete et al., 2025)] or in combination with CT [tDCS + CT in one meta-analysis (Chu and Cheng, 2025) and rTMS + CT in one meta-analysis (Liu et al., 2023)]. Moreover, Inagawa et al. (2019a) confirmed the effectiveness of tDCS in improving global cognition; however, their sample size was small, and the included participants had frontotemporal dementia or stroke. In general, the cognitive scales varied across the included RCTs. In the current study, we used common outcome measures—ADAS-Cog and MMSE scores—to clarify the effects of NIBS.

In the subgroup analysis of stimulation types, our meta-analysis revealed rTMS to have significant effects with respect to improving global cognition in patients with AD, as indicated by the MMSE (12 trials; SMD = 0.70, 95% CI = 0.23 to 1.17, *I*^2^ = 83%, *p* = 0.003) and the ADAS-Cog (8 trials; SMD = −0.32, 95% CI = −0.62 to −0.01, *I*^2^ = 53%, *p* = 0.04), as well as did tDCS, as indicated by the MMSE (9 trials; SMD = 0.45, 95% CI = 0.17 to 0.73, *I*^2^ = 28%, *p* = 0.002) but not the ADAS-Cog (2 trials; SMD = −0.46, 95% CI = −1.18 to 0.27, *I*^2^ = 62%, *p* = 0.22). This finding is consistent with those of previous research: rTMS^30^ and tDCS (Chen et al., 2022; Hou et al., 2024) can improve MMSE scores in patients with MCI and AD. Moreover, similar to the results of another meta-analysis (Chen et al., 2022), those of the current study revealed that ADAS-Cog scores did not indicate tDCS treatment to have a significant effect, although this result may be related to only a small number of trials including this scale.

The subgroup analysis of NIBS alone and in combination with CT revealed improvements in ADAS-Cog and MMSE scores; however, undergoing any adjunctive CT along with NIBS resulted in improved ADAS-Cog scores [in 3 trials (Andrade et al., 2022; Lee et al., 2016; Zhao et al., 2017) for mild-to-moderate AD]. A similar positive but nonsignificant trend was observed in MMSE scores [from 4 trials (Bagattini et al., 2020; Cotelli et al., 2014; Zhang et al., 2019; Zhao et al., 2017) for mild-to-moderate AD]. From these results, we could not conclude whether NIBS adjunctive CT effectively improves cognition in patients with mild-to-moderate AD. Regarding training delivery models (eg, home based), only 4 RCTs (Im et al., 2019; Kim and Yang, 2023; Meléndez et al., 2023; Satorres et al., 2023) involving tDCS (at 2 mA for 20–30 min over 5–7 days during a 1–24-week intervention period) reported the delivery model to have a positive effect on cognitive function.

In the included studies, 17 target areas were noted, with 5 single stimulation site types common to 14 (38%) studies: these sites were the left dorsolateral prefrontal cortex (DLPFC) (Ahmed et al., 2012; Bagattini et al., 2020; Cotelli et al., 2014; Fonte et al., 2024; Khedr et al., 2014; Li et al., 2023; Li et al., 2021; Lin et al., 2024; Wu et al., 2022; Wu et al., 2024), precuneus (Koch et al., 2025; Koch et al., 2022), medial prefrontal cortex (LoBue et al., 2025), lateral parietal lobule (Wei et al., 2022), and left parietal cortex (Jia et al., 2021). The most common site was the left DLPFC (9/37, 24%). Among the remaining studies (23/37, 62%), the most common site was the bilateral DLPFC (6/37, 16%) (Im et al., 2019; Kim and Yang, 2023; Moussavi et al., 2024; Saitoh et al., 2022; Wu et al., 2024; Zhou et al., 2022), followed by 6 other brain areas (3/37, 8%): the right and left DLPFC, Broca and Wernicke areas, and right and left parietal somatosensory association cortex (Andrade et al., 2022; Lee et al., 2016; Rabey et al., 1996). In general, 40% (15/37) of the analyzed studies included the left DLPFC as a stimulation site.

### 4.2 Intervention regimen characteristics (length, duration, frequency, and exposure times)

The duration of the cognitive effects following NIBS in patients with MCI and AD is a key concern in NIBS. In this study, subgroup analysis of the immediate effect of treatment revealed that MMSE scores were significantly higher in patients exposed to treatment for 2 weeks (Wang et al., 2024; Cotelli et al., 2014; Jia et al., 2021; Khedr et al., 2019; Lin et al., 2024; Satorres et al., 2023; Wei et al., 2022; Wu et al., 2022; Wu et al., 2024), 4 weeks (Bagattini et al., 2020; Hu et al., 2022; Qin et al., 2023; Yao et al., 2022; Zhang et al., 2019); moreover, the ADAS-Cog scores were significantly lower in patients who received treatment for 6 weeks (Lee et al., 2016; Li et al., 2023; Li et al., 2021; Zhao et al., 2017) or longer (Andrade et al., 2022; Koch et al., 2022; Leocani et al., 2020). Two previous meta-analyses have revealed that cognitive function continues to improve after intervention ends, with the effects lasting for >8 weeks according to MMSE scores (Hou et al., 2024) and approximately 12 weeks according to ADAS-Cog (Liu et al., 2023). Both of these studies retrieved full texts published in English and Chinese. Hou et al. (2024) included participants with MCI, whereas Liu et al. (2023) explored the effects of rTMS combined with CT. In general, differences in neuropsychological scales, disease severity and stages, and NIBS treatments can lead to studies reporting different outcomes.

In the current study, the cognitive improvements, as indicated by higher MMSE scores, were maintained for 4 to 8 weeks after the completion of NIBS. However, the positive effects of NIBS on cognitive function, measured using the ADAS-Cog, were not sustained over a longer follow-up period. However, the current results based on ADAS-Cog scores should be interpreted with caution because very few RCTs included in this study used the scale. Overall, NIBS demonstrated short-term cognitive benefits. Improvements in MMSE scores were observed following 2–4 weeks of intervention, whereas improvements in ADAS-Cog scores were observed following interventions lasting at least 6 weeks. In addition, improvements measured by the MMSE were maintained for 4–8 weeks after completion of the intervention.

We used meta-regression to evaluate the impact of 6 intervention time variables on NIBS outcomes. Our results demonstrated that none of the 6 intervention time variables had a significant effect on cognitive function. However, analysis of the ADAS-Cog scores of the 6 intervention time variables revealed that total intervention time (*β* = −0.061, *Q* = 7.88, df = 1, *p* = 0.005) and total intervention sessions (β = −0.033, *Q* = 3.92, df = 1, *p* = 0.048) mitigated the degree of cognitive impairment. The ADAS-Cog demonstrated increased sensitivity to the effects of total NIBS intervention time (weeks) and total NIBS intervention sessions (sessions × weeks), whereas the MMSE was constrained by ceiling effects, limited domain coverage, and measurement noise. This study identified four common administration regimens across the included studies: (1) 5 to 7 consecutive days per week, (2) a 2-week intensive intervention phase followed by a 22- to 50-week maintenance phase, (3) two sessions per day, with intervention durations ranging from 1 to 6 weeks depending on the study protocol; and (4) 3 sessions per week. Moreover, 8 weekly exposure times were observed: 10, 15, 20, 25, 30, 45, 50, and 60 min. In general, NIBS was administered in 20-min sessions over 5 to 7 consecutive days per week.

### 4.3 Sample characteristics (disease degree and medicine situation)

Regarding disease severity, subgroup analysis based on MMSE outcomes revealed that NIBS had significant and positive effects in patients with aMCI–mild AD (4 trials; SMD = 0.54, 95% CI = 0.03 to 1.04, *I*^2^ = 31%, *p* = 0.04) and mild-to-moderate AD (16 trials; SMD = 0.41, 95% CI = 0.20 to 0.62, *I*^2^ = 40%, *p* < 0.001). In patients with moderate AD, these effects were not significant (2 trials, MD = 1.33, 95% CI = −0.03 to 2.69, *I*^2^ = 91%, *p* = 0.06). Subgroup analysis based on ADAS-Cog outcomes also revealed significant improvements in patients with mild-to-moderate AD (8 trials, MD = −0.50, 95% CI = −0.89 to −0.10, *I*^2^ = 74%, *p* = 0.01) and moderate AD (2 trials, MD = −0.36, 95% CI = −0.70 to −0.02, I2 = 0%, p = 0.04), with negative values indicating improvements in cognitive performance. However, comparisons across different disease severity levels revealed no significant differences in treatment effects, as indicated by MMSE outcomes. Although improvements were observed in the ADAS-Cog scores, no significant differences in effect size were observed between different disease severity groups. A meta-analysis reported effect sizes for rTMS that were significant when estimated using both the MMSE and ADAS-Cog in patients with MCI (Chou et al., 2020; Wang et al., 2020) and AD (Chou et al., 2020). In another study, the effects of tDCS were greater in patients with AD (Chen et al., 2022) according to MMSE scores; in yet another study, the effects of NIBS were greater in patients with AD according to both the MMSE and the ADAS-Cog (Teselink et al., 2021). However, the data on MCI were limited and incomplete in the present study. Therefore, further research is required to examine the effectiveness of NIBS in individuals with MCI, and our findings should be interpreted with caution.

To the best of our knowledge, no previous study has evaluated whether stable medication status influences the effects of NIBS in patients with AD. Our meta-regression indicated that a longer duration of pharmacological treatment was associated with diminished treatment effects of NIBS. Consistent with this finding, in our subgroup analysis, greater improvements were observed in participants who received no pharmacological treatment or had been on stable pharmacological treatment for ≤3 months, among those with AD. These findings suggest that earlier implementation of NIBS, before prolonged exposure to pharmacological treatment, may maximize its therapeutic benefits.

### 4.4 Limitations

This study has several limitations. First, although 37 RCTs were identified, 14 of these trials had small sample sizes (*n* ≤ 30), which may have limited the study’s statistical power, particularly in subgroup analyses. Second, subgroup analyses by geographic region and NIBS modality were conducted as an exploratory (*post hoc*) approach to explore potential sources of heterogeneity. Therefore, their results should be interpreted with caution. Third, several potentially important stimulation-related factors could not be adequately examined. The effects of stimulation site could not be fully evaluated because of the wide variation in target regions and the limited number of studies within individual stimulation-site categories. Moreover, participant-specific anatomical characteristics, such as cortical atrophy, scalp-to-cortex distance, and brain morphology, were generally not reported in the included studies. Consequently, the potential influence of these factors on treatment response could not be investigated. Future studies incorporating individual MRI data and computational electric-field modeling may provide a better understanding of how anatomical variability influences NIBS outcomes and may help explain part of the observed heterogeneity. Fourth, some variables contained a large number of categories, complicating the analysis of factors such as birth generation and session length. Finally, some eligible RCTs did not report mean scores in the relative outcomes, or their corresponding authors did not offer to provide data via email; these RCTs could not be included in our meta-analysis.

## 5 Conclusion

In this meta-analysis, we evaluated the effects of NIBS on the cognitive function of individuals with AD and MCI. Our findings suggest that NIBS, particularly rTMS, is associated with modest improvements in global cognition, as measured using the MMSE and ADAS-Cog. Cognitive benefits were observed after short-term interventions (2–6 weeks), and the improvements achieved at the end of treatment were maintained during follow-up at 4–8 weeks, as confirmed by MMSE outcomes. In addition, Pharmacological treatment might associate with diminished treatment effects of NIBS. Because of the moderate heterogeneity observed across studies and the exploratory nature of several subgroup analyses, these findings should be interpreted with caution.

## Data Availability

The original contributions presented in the study are included in the article/Supplementary material, further inquiries can be directed to the corresponding author.
