# Supplementary material for: Effectiveness of noninvasive brain stimulation techniques on cognitive function in individuals with Alzheimer’s disease and MCI: a systematic review and meta-analysis
Source: Front Aging Neurosci. 2026 Jul 27;18:1782521. doi: 10.3389/fnagi.2026.1782521 (PMC13454334; doi:10.3389/fnagi.2026.1782521)
Supplement: Supplementary file 1 [file Supplementary_file_1.docx]

# Supplement 1

Table 1. Search Strategy Table

| **Cochrane Library search strategy –last searched on Jun 22, 2025** | | |
| --- | --- | --- |
| #1 | (Alzheimer*):ti,ab,kw OR (dement*):ti,ab,kw | 27,649 |
| #2 | (transcranial):ti,ab,kw OR (TMS):ti,ab,kw OR (rTMS):ti,ab,kw OR (tDCS):ti,ab,kw OR (tES):ti,ab,kw | 20,817 |
| #3 | (random*):ti,ab,kw OR (RCT):ti,ab,kw | 1,405,599 |
| #4 | #1 AND #2 AND #3 | 633 |

| **Web of Science search strategy –last searched on Jun 22, 2025** | | **Results** |
| --- | --- | --- |
| **1** | Alzheimer* (Topic) OR dement* (Topic) | 417,023 |
| **2** | transcranial (Topic) OR TMS (Topic) OR rTMS (Topic) OR tDCS (Topic) OR tES | 87,711 |
| **3** | random* (Topic) OR RCT | 2,280,438 |
| **4** | **#1 AND #2 AND #3** | 437 |

| **PubMed search strategy –last searched on Jun 22, 2025** | | **Results** |
| --- | --- | --- |
| #1 | Search: ((Alzheimer*[Title/Abstract])) OR (dement*[Title/Abstract]) | 323,534 |
| #2 | Search: ((((transcranial[Title/Abstract]) OR (TMS[Title/Abstract])) OR (rTMS[Title/Abstract])) OR (tDCS[Title/Abstract])) OR (tES[Title/Abstract]) | 63,076 |
| #3 | Search: (random*[Title/Abstract]) OR (RCT[Title/Abstract]) | 1,651,198 |
| #4 | #1 AND #2 AND #3 | 323 |

| **Scopus search strategy –last searched on Jun 22, 2025** | | **Results** |
| --- | --- | --- |
| **1** | ( TITLE-ABS-KEY ( alzheimer* ) OR TITLE-ABS-KEY ( dement* ) ) | 491,084 |
| **2** | ( TITLE-ABS-KEY ( transcranial ) OR TITLE-ABS-KEY (TMS ) OR TITLE-ABS-KEY (rTMS ) OR TITLE-ABS-KEY (tDCS ) OR TITLE-ABS-KEY (tES ) ) | 117,274 |
| **3** | ( TITLE-ABS-KEY ( random* ) OR TITLE-ABS-KEY (RCT ) ) | 3,618,304 |
| **4** | **#1 AND #2 AND #3** | 566 |

Table 2 Articles Excluded Reason

| **Author, year** | **Title** | **excluded reason** |
| --- | --- | --- |
| Alcalá-Lozano,2018 | Similar clinical improvement and maintenance after rTMS at 5 Hz using a simple vs. complex protocol in Alzheimer's disease | NIBS in both groups |
| Anderkova, 2015 | Distinct Pattern of Gray Matter Atrophy in Mild Alzheimer's Disease Impacts on Cognitive Outcomes of Noninvasive Brain Stimulation | NIBS in both groups |
| André, 2016 | At-home tDCS of the left dorsolateral prefrontal cortex improves visual short-term memory in mild vascular dementia | Non-AD participants (other dementias) |
| Assogna, 2021 | Gamma-induction in frontotemporal dementia (GIFTeD) randomized placebo-controlled trial: rationale, noninvasive brain stimulation protocol, and study design | Non-AD participants (other dementias) |
| Benussi, 2020 | Transcranial stimulation in frontotemporal dementia: a randomized, double-blind, sham-controlled trial | Non-AD participants (other dementias) |
| Benussi, 2021 | Exposure to gamma tACS in Alzheimer's disease: a randomized, double-blind, sham-controlled, crossover, pilot study | lacked usable data |
| Benussi, 2022 | Increasing Brain Gamma Activity Improves Episodic Memory and Restores Cholinergic Dysfunction in Alzheimer's Disease | Duplicate studies from the same research group |
| Benussi, 2024 | Alpha tACS Improves Cognition and Modulates Neurotransmission in Dementia with Lewy Bodies | Non-AD participants (other dementias) |
| Boggio, 2009 | Temporal cortex direct current stimulation enhances performance on a Visual recognition memory task in Alzheimer disease | NIBS in both groups |
| Bystad, 2016 | Transcranial direct current stimulation as a memory enhancer in patients with Alzheimer's disease: a randomized, placebo-controlled trial | Non-AD participants (healthy ) |
| Cotelli, 2011 | Improved language performance in Alzheimer disease following brain stimulation | NIBS in both groups |
| Eliasova, 2014 | Non-invasive brain stimulation of the right inferior frontal gyrus may improve attention in early Alzheimer's disease: a pilot study | NIBS in both groups |
| Gangemi, 2021 | Effects of short- and long-term neurostimulation (tDCS) on Alzheimer's disease patients: two randomized studies | NIBS in both groups |
| Hoy, 2023 | Gamma connectivity predicts response to intermittent theta burst stimulation in Alzheimer's disease: a randomized controlled trial | lacked usable data |
| Khedr, 2020 | The effect of repetitive transcranial magnetic stimulation on cognitive impairment in Parkinson's disease with dementia: pilot study | Non-AD participants (other dementias) |
| Kumar, 2023 | Effects of repetitive paired associative stimulation on brain plasticity and working memory in Alzheimer's disease: a pilot randomized double-blind-controlled trial | lacked usable data |
| Lane, 2023 | Adjunctive transcranial direct current stimulation (tDCS) plus sodium benzoate for the treatment of early-phase Alzheimer's disease: a randomized, double-blind, placebo-controlled trial | combined with medication |
| Lithgow, 2025 | Surface Versus Penetrative rTMS Stimulation May Be More Effective for AD Patients with Cerebrovascular Disease | Non-AD participants (other dementias) |
| Lu, 2025 | Pre-treatment subjective sleep quality as a predictive biomarker of tDCS effects in preclinical Alzheimer's disease patients: secondary analysis of a randomised clinical trial | unclear procedure |
| Mencarelli, 2024 | Macro and micro structural preservation of grey matter integrity after 24 weeks of rTMS in Alzheimer's disease patients: a pilot study | lacked usable data |
| Ownby, 2021 | Computer-Delivered Cognitive Training and Transcranial Direct Current Stimulation in Patients With HIV-Associated Neurocognitive Disorder: a Randomized Trial | Non-AD participants (other dementias) |
| Park, 2024 | Efficacy of Home-Based Remotely Supervised Transcranial Direct Current Stimulation for Managing Neuropsychiatric Symptoms in Older Adults With Alzheimer's Disease and Related Dementias | lacked usable data |
| Qin, 2022 | Effects of repetitive transcranial magnetic stimulation combined with cognitive training on resting-state brain activity in Alzheimer's disease | Duplicate studies from the same research group |
| Rektorova, 2005 | Cognitive functioning after repetitive transcranial magnetic stimulation in patients with cerebrovascular disease without dementia: a pilot study of seven patients | Non-AD participants (other dementias) |
| Roncero, 2017 | Inferior parietal transcranial direct current stimulation with training improves cognition in anomic Alzheimer's disease and frontotemporal dementia | Non-AD participants (other dementias) |
| Sedlackova, 2008 | Neurocognitive effects of repetitive transcranial magnetic stimulation in patients with cerebrovascular disease without dementia | Non-AD participants (other dementias) |
| Smirni, 2021 | Verbal Fluency in Mild Alzheimer's Disease: transcranial Direct Current Stimulation over the Dorsolateral Prefrontal Cortex | unclear procedure |
| Teti Mayer, 2021 | Repetitive transcranial magnetic stimulation as an add-on treatment for cognitive impairment in alzheimer’s disease and its impact on self-rated quality of life and caregiver’s burden | Not an RCT |
| Varastegan, 2023 | Remember NIBS? tACS improves memory performance in elders with subjective memory complaints | Non-AD participants (other dementias) |
| Wei, 2021 | Repetitive Transcranial Magnetic Stimulation for Alzheimer's Disease Based on Apolipoprotein E Genotyping: protocol for a Randomized Controlled Study | NIBS in both groups |
| Zhang, 2022 | Evaluating the treatment outcomes of repetitive transcranial magnetic stimulation in patients with moderate-to-severe Alzheimer's disease | lacked usable data |

**Figure 1 funnel plot.** The funnel plots revealed symmetry in the assessments of cognitive function on the MMSE (*P* = .43, Egger test; *P* = .22, Begg test) and for ADAS-Cog outcomes (*P* = .77, Egger test; *P* = .55, Begg test).

# Supplement 2

MMSE_endpoint

(1) NIBS technique singularly or in combination with CT for individuals with dementia

Fig 1a NIBS technique singularly on the MMSE.

Fig 1b NIBS technique combination with CT or not on the MMSE.

(2) duration of efficacy on global cognition

Fig 1c NIBS intervention period on the MMSE.

(3) identify the optimal treatment protocol considering different intervention parameters on cognitive function.

Fig 1d population characteristic on the MMSE.

Fig 1e stable medication period on the MMSE.

(4) NIBS effects across dementia severity levels.

Fig 1f effects of dementia severity on the MMSE.

ADAS-Cog_endpoint

(1) NIBS technique singularly or in combination with CT for individuals with dementia

Fig 2a NIBS technique singularly on the ADAS-Cog outcome.

Fig 2b NIBS technique combination with CT or not on the ADAS-Cog outcome.

(2) duration of efficacy on global cognition

Fig 2c NIBS intervention period on the ADAS-Cog outcome.

(3) identify the optimal treatment protocol considering different intervention parameters on cognitive function.

Fig 2d population characteristic on the ADAS-Cog outcome.

Fig 2e stable medication period on the ADAS-Cog outcome.

(4) NIBS effects across dementia severity levels.

Fig 2f effects of dementia severity on the ADAS-Cog outcome.

MMSE_follow-up

(1) NIBS technique singularly or in combination with CT for individuals with dementia

Fig 3a NIBS technique singularly on the MMSE during follow-up.

Fig 3b NIBS technique combination with CT or not on the MMSE during follow-up.

(2) follow-up period of efficacy on global cognition

Fig 3c NIBS follow-up period on the MMSE during follow-up.

(3) identify the optimal treatment protocol considering different intervention parameters on cognitive function.

Fig 3d population characteristic on the MMSE during follow-up.

Fig 3e stable medication period on the MMSE during follow-up.

ADAS-Cog_follow-up

(1) NIBS technique singularly or in combination with CT for individuals with dementia

Fig 4a NIBS technique singularly on the ADAS-Cog during follow-up.

Fig 4b NIBS technique combination with CT or not on the ADAS-Cog during follow-up.

(2) follow-up period of efficacy on global cognition

Fig 4c NIBS follow-up period on the ADAS-Cog during follow-up.

(3) identify the optimal treatment protocol considering different intervention parameters on cognitive function.

Fig 4d population characteristic on the ADAS-Cog during follow-up.

Fig 4e stable medication period on the ADAS-Cog during follow-up.

**Table 1a Primary Outcome.**

| **Outcome** | **Studies** | **Participants** | **Statistical Method** | **Effect Estimate** |
| --- | --- | --- | --- | --- |
| 1.1 MMSE_ | 27 | 1007 | Std. Mean Difference (IV, Random, 95% CI) | 0.58 [0.32, 0.84] |
| 2.1 MMSE_follow up_ | 17 | 514 | Std. Mean Difference (IV, Random, 95% CI) | 0.56 [0.24, 0.88] |
| 3.1 ADAS-cog_ | 14 | 676 | Std. Mean Difference (IV, Random, 95% CI) | -2.01 [-3.29, -0.73] |
| 4.1 ADAS-cog follow up_ | 11 | 474 | Std. Mean Difference (IV, Random, 95% CI) | -0.25 [-0.52, 0.01] |

**Table 1b Subgroup Analysis for Primary Outcome.**

|  | MMSE_endpoint | | | | ADAS-Cog_endpoint | | | |
| --- | --- | --- | --- | --- | --- | --- | --- | --- |
|  | **Outcome or Subgroup** | **Studies** | **Participants** | **Std. Mean Difference**  **(IV, Random, 95% CI)**  **Effect Estimate** | **Outcome or Subgroup** | **Studies** | **Participants** | **Std. Mean Difference**  **(IV, Random, 95% CI)**  **Effect Estimate** |
| NIBS technique singularly or in combination with CT for individuals with dementia | 1.2 NIBS technique singularly |  |  |  | 2.2 NIBS technique singularly |  |  |  |
|  | 1.2.1 rTMS | 13 | 498 | **0.70 [0.23, 1.17]** | 2.2.1 rTMS | 9 | 418 | -0.32 [-0.62, -0.01] |
|  | 1.2.2 tDCS | 10 | 347 | **0.45 [0.17, 0.73]** | 2.2.2 tDCS | 2 | 152 | -0.46 [-1.18, 0.27] |
|  | 1.2.3 rTMS+tDCS | 1 | 28 | 1.17 [0.25, 2.08] | 2.2.3 rTMS+tDCS | 1 | 28 | -0.41 [-1.28, 0.45] |
|  | 1.2.4 iBTS | 3 | 134 | 0.33 [-0.28, 0.94] |  |  |  |  |
|  | 1.3 NIBS technique combination with CT or others |  |  |  | 2.3 NIBS technique combination with CT or others |  |  |  |
|  | 1.3.1 Combined with CT | 4 | 126 | 0.50 [-0.18, 1.17] | 2.3.1 Combined with CT | 3 | 92 | -0.75 [-1.64, 0.14] |
|  | 1.3.2 Combined with other | 2 | 46 | 0.70 [-0.24, 1.65] | 2.3.2 Combined with other | 1 | 28 | -0.41 [-1.28, 0.45] |
|  | 1.3.3 Not | 21 | 835 | **0.59 [0.28, 0.89]** | 2.3.3 Not | 9 | 514 | -0.35 [-0.67, -0.03] |
| intervention / follow-up period | 1.4 intervention period |  |  |  | 2.4 intervention period |  |  |  |
|  | 1.4.1 1wk | 3 | 63 | 0.47 [-0.06, 0.99] | 2.4.1 1wk |  |  |  |
|  | 1.4.2 2wk | 10 | 402 | **0.35 [0.06, 0.63]** | 2.4.2 2wk | 1 | 77 | 0.23 [-0.25, 0.71] |
|  | 1.4.3 4wk | 7 | 203 | **0.78 [0.46, 1.10]** | 2.4.3 4wk | 5 | 188 | -0.10 [-0.43, 0.22] |
|  | 1.4.4 6wk | 5 | 273 | 0.74 [-0.39, 1.88] | 2.4.4 6wk | 4 | 255 | **-0.58 [-0.83, -0.33]** |
|  | 1.4.5 >6wk | 2 | 66 | 0.74 [-0.05, 1.53] | 2.4.5 >6wk | 3 | 114 | **-1.03 [-1.60, -0.45]** |
| population characteristic (Western ethnicity or not, stable medication period) | 1.5 Western and non-Western |  |  |  | 2.5 Western and non-Western |  |  |  |
|  | 1.5.1 Western | 6 | 187 | **0.43 [0.13, 0.72]** | 2.5.1 Western ethnicity | 4 | 234 | -0.23 [-0.82, 0.35] |
|  | 1.5.2 non-Western | 21 | 820 | **0.64 [0.32, 0.96]** | 2.5.2 non-Western | 9 | 400 | **-0.57 [-0.85, -0.29]** |
|  | 1.6 stable medication period |  |  |  | 2.6 stable medication period |  |  |  |
|  | 1.6.1 used <=3m | 13 | 464 | **0.52 [0.24, 0.80]** | 2.6.1 used 3m | 8 | 321 | -0.08 [-0.33, 0.18] |
|  | 1.6.2 used 6m | 3 | 86 | 0.24 [-0.21, 0.68] | 2.6.2 used 6m | 1 | 50 | **-0.98 [-1.57, -0.39]** |
|  | 1.6.3 uncertain | 8 | 379 | **0.81 [0.11, 1.50]** | 2.6.3 uncertain | 3 | 227 | **-0.62 [-0.89, -0.36]** |
|  | 1.6.4 none_use | 3 | 78 | **0.61 [0.14, 1.08]** |  |  |  |  |

**Table 2 Subgroup Analysis for Secondary Outcome.**

|  | MMSE_follow-up | | | | ADAS-Cog_follow-up | | | |
| --- | --- | --- | --- | --- | --- | --- | --- | --- |
|  | **Outcome or Subgroup** | **Studies** | **Participants** | **Std. Mean Difference**  **(IV, Random, 95% CI)**  **Effect Estimate** | **Outcome or Subgroup** | **Studies** | **Participants** | **Std. Mean Difference**  **(IV, Random, 95% CI)**  **Effect Estimate** |
| NIBS technique singularly or in combination with CT for individuals with dementia | 1.2 NIBS technique singularly |  |  |  | 2.2 NIBS technique singularly |  |  |  |
|  | 1.2.1 rTMS | 11 | 364 | **0.56 [0.24, 0.88]** | 2.2.1 rTMS | 9 | 418 | -0.29 [-0.59, 0.01] |
|  | 1.2.2 tDCS | 4 | 78 | **0.63 [0.19, 1.07]** | 2.2.2 tDCS | 1 | 28 | 0.10 [-0.76, 0.95] |
|  | 1.2.3 rTMS+tDCS | 1 | 28 | 0.32 [-0.23, 0.87] | 2.2.3 rTMS+tDCS | 1 | 28 | -0.27 [-1.13, 0.59] |
|  | 1.2.4 iBTS | 1 | 44 | 0.22 [-0.37, 0.81] |  |  |  |  |
|  | 1.3 NIBS technique combination with CT or others |  |  |  | 2.3 NIBS technique combination with CT or others |  |  |  |
|  | 1.3.1 Combined with CT | 5 | 148 | 0.28 [-0.18, 0.73] | 2.3.1 Combined with CT | 2 | 56 | -0.40 [-0.95, 0.16] |
|  | 1.3.2 Combined with other | 2 | 46 | 0.66 [0.00, 1.33] | 2.3.2 Combined with other | 1 | 28 | -0.27 [-1.13, 0.59] |
|  | 1.3.3 Not | 10 | 320 | **0.69 [0.22, 1.16]** | 2.3.3 Not | 8 | 390 | -0.24 [-0.57, 0.10] |
| intervention / follow-up period | 1.4 follow-up period |  |  |  | 2.4 follow-up period |  |  |  |
|  | 1.4.1 4wk | 3 | 62 | **0.93 [0.39, 1.46]** | 2.4.1 4wk |  |  |  |
|  | 1.4.2 6wk | 2 | 56 | 0.04 [-0.52, 0.60] | 2.4.2 6wk | 2 | 56 | -0.40 [-0.95, 0.16] |
|  | 1.4.3 8wk | 6 | 203 | **0.47 [0.17, 0.77]** | 2.4.3 8wk | 4 | 109 | -0.26 [-0.68, 0.17] |
|  | 1.4.4 12wk | 4 | 161 | 0.86 [-0.19, 1.91] | 2.4.4 12wk | 1 | 75 | 0.01 [-0.44, 0.47] |
|  | 1.4.5 24wk | 3 | 32 | -0.00 [-0.74, 0.74] | 2.4.5 16wk | 1 | 28 | -0.37 [-1.12, 0.39] |
| population characteristic (Western ethnicity or not, stable medication period) |  |  |  |  | 2.4.5 24wk | 1 | 50 | -1.17 [-1.78, -0.57] |
|  |  |  |  |  | 2.4.5 28wk | 2 | 156 | 0.07 [-0.26, 0.41] |
|  | 1.5 Western and non-Western |  |  |  | 2.5 Western and non-Western |  |  |  |
|  | 1.5.1 Western ethnicity | 4 | 100 | 0.39 [-0.09, 0.86] | 2.5.1 Western ethnicity | 4 | 234 | -0.32 [-0.92, 0.27] |
|  | 1.5.2 non-Western | 13 | 414 | **0.61 [0.22, 1.00]** | 2.5.2 non-Western | 7 | 240 | -0.19 [-0.46, 0.08] |
|  | 1.6 stable medication period |  |  |  | 2.6 stable medication period |  |  |  |
|  | 1.6.1 used 3m | 8 | 247 | **0.54 [0.27, 0.81]** | 2.6.1 used 3m | 6 | 265 | -0.05 [-0.32, 0.21] |
|  | 1.6.2 used <3m | 2 | 56 | 0.04 [-0.52, 0.60] | 2.6.2 used <3m | 2 | 56 | -0.40 [-0.95, 0.16] |
|  | 1.6.3 used 6m | 2 | 32 | -0.00 [-0.74, 0.74] | 2.6.3 used 6m | 1 | 50 | -1.17 [-1.78, -0.57] |
|  | 1.6.4 uncertain | 3 | 134 | 0.98 [-0.39, 2.35] | 2.6.4 uncertain | 2 | 103 | -0.09 [-0.48, 0.30] |
|  | 1.6.5 none_use | 2 | 45 | **0.75 [0.04, 1.46]** |  |  |  |  |

# Supplement 3

Fig 1a leave-one-out meta-analysis of MMSE endpoint outcome

Fig 1b leave-one-out meta-analysis of ADAS-Cog endpoint outcome

Fig 2 stable medication period

Table 1 Meta-Regression Analyses for MMSE Endpoint, ADAS-Cog Endpoint.

|  | MMSE_endpoint | | ADAS-Cog_endpoint | |
| --- | --- | --- | --- | --- |
| variables | Coefficient(SE) | *P* | Coefficient(SE) | *P* |
| session length (min) | -0.02 | 0.1154 | 0.0069 | 0.6378 |
| weekly frequency (times) | -0.0494 | 0.3765 | 0.0549 | 0.4206 |
| total duration (week) | -0.0017 | 0.9453 | **-0.0608** | **0.005** |
| total exposure (min) | -0.0001 | 0.5474 | -0.0001 | 0.6851 |
| total sessions (day) | -0.0001 | 0.9757 | **-0.0331** | **0.0476** |
| weekly exposure (min) | -0.0016 | 0.1244 | 0.0022 | 0.3211 |
| follow-up (week) |  |  |  |  |
| NIBS_rTMS^a^ | 0.9435 | 0.2208 |  |  |
| NIBS_rTMS_CT^b^ | 1.0736 | 0.2144 | 0.1082 | 0.8249 |
| NIBS_tDCS^c^ | 0.8275 | 0.2898 | -0.3579 | 0.3665 |
| NIBS_tDCS_CT^d^ | -0.155 | 0.8887 |  |  |
| NIBS_tDCS_motor^e^ | 0.4542 | 0.6814 |  |  |
| NIBS_tDCS_rTMS^f^ | 1.4424 | 0.1859 | 0.0184 | 0.9785 |
| female (percent) | -0.052 | 0.9696 | -0.8141 | 0.713 |
| NIBS_CT^g^ | -0.1 | 0.7578 | 0.1945 | 0.6732 |
|  |  |  |  |  |
|  |  |  |  |  |
| Stepwise regression |  |  |  |  |
| Constant | Not significant | |  | |
| total duration (week) |  |  | **-0.0608** | **0.005** |
| Adjusted R^2^ (%) |  |  | 0.55 |  |
| 95%CI |  |  | [-0.1033, -0.0184] |  |
| Q |  |  | 788 |  |
| I^2^ Residual (%) |  |  | 47.3 |  |

Notes.^a^1 = rTMS, 0 = Not; ^b^1 = rTMS + CT, 0 = Not; ^c^1 = tDCS, 0 = Not; ^d^1 = tDCS + CT, 0 = Not; ^e^1 = tDCS + motor, 0 = Not; ^f^1 = tDCS + rTMS, 0 = Not; ^g^1 = Cognitive Training (CT), 0 = Not.
